# Integrative Multi‐Omics Analysis Reveals a Mitochondrial–Immune Axis Associated With Neoadjuvant Chemotherapy Response in High‐Grade Serous Ovarian Cancer

**DOI:** 10.1002/advs.202523029

**Published:** 2026-07-20

**Authors:** Wei Jiang, Yi Wang, Xiaohang Lu, Qianlan Yao, Libing Xiang, Xu Cai, Xiaoyu Tu, Shaoxian Tang, Ying Xu, Yufan Cheng, Liming Shen, Huijuan Yang

**Affiliations:** ^1^ Department of Gynecological Oncology Fudan University Shanghai Cancer Center Fudan University Shanghai China; ^2^ Department of Oncology Shanghai Medical College Fudan University Shanghai China; ^3^ Department of Pathology Oncology Fudan University Shanghai Cancer Center Fudan University Shanghai China; ^4^ Institute of Pathology Fudan University Shanghai China; ^5^ Department of Gynecologic Oncology Zhongshan Hospital Fudan University Shanghai China; ^6^ School of Medicine Southern University of Science and Technology Shenzhen China; ^7^ College of Life Science and Oceanography Shenzhen University Shenzhen China

**Keywords:** CD19^+^ B cells, high‐grade serous ovarian cancer, multi‐omics analysis, NDUFA8, neoadjuvant chemotherapy response

## Abstract

Neoadjuvant chemotherapy (NACT) is a standard treatment for high‐grade serous ovarian cancer (HGSOC), yet determinants of therapeutic response remain incompletely defined. Here, multi‐omics analysis of 102 tumor samples revealed that NACT reshapes the tumor microenvironment, enhancing B‐ and NK‐cell infiltration and activating antigen presentation and BCR/TCR signaling pathways. Notably, Nab‐paclitaxel outperformed paclitaxel in promoting B cell‐mediated humoral immunity. Unsupervised clustering identifies two biologically distinct subtypes: an immune‐active chemotherapy response score (CRS)‐high subtype characterized by CD19^+^ B‐cell enrichment and complement activation, and a metabolically rewired CRS‐low subtype exhibiting heightened oxidative phosphorylation (OXPHOS) and mitochondrial remodeling. Platinum‐resistant tumors display pronounced OXPHOS dependency, indicating a bioenergetic vulnerability. Pharmacologic inhibition of mitochondrial Complex I with IACS‐010759, or genetic silencing of its core subunit NDUFA8, selectively restores carboplatin sensitivity in resistant cells and xenograft models. Complex I disruption suppresses mitochondrial respiration, induces membrane depolarization and oxidative stress, and triggers stress‐adaptive mitophagy, thereby lowering the threshold for platinum‐induced cytotoxic stress. Clinically, CD19^+^ B‐cell infiltration and NDUFA8 expression are significantly associated with platinum responsiveness and NACT outcome. Together, these findings define immune activation and mitochondrial bioenergetic dependency as dual determinants of chemotherapy response and highlight Complex I–targeted intervention as a rational strategy to overcome platinum resistance in HGSOC.

## Graphical abstract

Integrative multi‐omics analysis delineates a mitochondrial–immune axis governing neoadjuvant chemotherapy response in high‐grade serous ovarian cancer. Immune‐active tumors exhibit enhanced B‐cell infiltration and favorable sensitivity, whereas metabolically rewired tumors display oxidative phosphorylation dependency and resistance. Targeting mitochondrial Complex I restores platinum responsiveness, uncovering bioenergetic–immune coupling as a mechanistically actionable vulnerability.

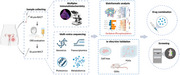

## 1 Introduction

Ovarian cancer is the most aggressive gynecological malignancy. In 2022, there were approximately 324 398 new cases and 206 839 deaths globally [1]. High‐grade serous ovarian cancer (HGSOC), which accounts for 70%–80% of ovarian malignancies, is often diagnosed at an advanced stage with disseminated peritoneal involvement. Primary debulking surgery (PDS) remains the cornerstone of initial management, while neoadjuvant chemotherapy (NACT) followed by interval debulking surgery (IDS) has emerged as a viable alternative first‐line treatment. The adoption of NACT has risen substantially over time, increasing from 8.6% to 22.6% between 2004 and 2013 [2] and from 17.6% to 45.1% between 2006 and 2016 for advanced ovarian cancer [3]. Nevertheless, the survival benefit of PDS versus NACT/IDS for newly diagnosed advanced ovarian cancer remains controversial. The EORTC 55971 and CHORUS trials demonstrated non‐inferiority of NACT relative to PDS in terms of progression‐free survival (PFS) and overall survival (OS)—along with benefits such as reduced surgical morbidity and improved quality of life [4, 5, 6]. More recently, the TRUST study, first presented at the 2025 ASCO Annual Meeting, showed that patients in good physical condition may achieve longer PFS and a trend toward improved OS with PDS compared to NACT/IDS, although this approach requires a high level of surgical expertise.

Thus, patient selection for optimal treatment is crucial in the management of advanced ovarian cancer. Current clinical decision‐making relies first on tumor burden and resectability. Accordingly, neoadjuvant chemotherapy (NACT) is preferred for FIGO stage IV patients, whereas those with initial abdominal disease burden <5 cm may have superior survival with PDS [7]. To objectively evaluate resectability, several scoring systems—such as the Suidan score, Fagotti score, and Peritoneal Carcinomatosis Index (PCI)—are widely used. Preoperative risk assessment has expanded to include patient‐specific factors, such as nutritional status, functional performance, and age, as exemplified by the Mayo classification algorithm and other comprehensive risk‐based tools developed to identify high‐risk patients more suitable for NACT [8]. However, although these models incorporate increasingly refined clinical parameters, they operate predominantly at the macroscopic level and share a fundamental limitation: none account for the intrinsic chemosensitivity of the tumor itself. Certain biomarkers, such as the Chemotherapy Response Score (CRS) [9] and the KELIM score [10], have been used to evaluate response to NACT, they face significant limitations. Both CRS and the KELIM score depend on histopathological evaluation of tumor tissues via H&E staining or serial CA‐125 measurements—meaning they can only be applied during or after NACT, not before initial treatment decisions. Moreover, despite growing recognition of the molecular heterogeneity of high‐grade serous ovarian cancer, no validated predictive models currently exist to reliably identify which patients will truly benefit from NACT prior to therapy initiation.

The current study, based on a clinical trial (ChiCTR1900026893) [11], investigated a cohort of 102 pre‐ and post‐NACT tumors by multi‐omics sequencing, including DNA sequencing, RNA sequencing, proteomics, and metabolomics sequencing. It demonstrated that NACT activated an immune state and impaired mitochondrial energy metabolism. Molecular stratification identifies an immune‐enriched subtype with abundant CD19^+^ B‐cell infiltration associated with favorable therapeutic response, and a metabolically rewired subtype marked by heightened oxidative phosphorylation linked to poor response. Mechanistically, platinum‐resistant tumors exhibit dependency on mitochondrial Complex I–mediated bioenergetic stability. Pharmacologic inhibition or genetic disruption of Complex I restores carboplatin sensitivity in resistant ovarian cancer cells and xenograft models. Collectively, these findings delineate immune activation and mitochondrial metabolic dependency as key molecular features associated with NACT responsiveness and highlight Complex I–targeted intervention as a potential chemosensitization strategy in high‐grade serous ovarian cancer.

## 2 Results

### 2.1 Patient Characteristics and Genomic Landscape

**FIGURE 1:**
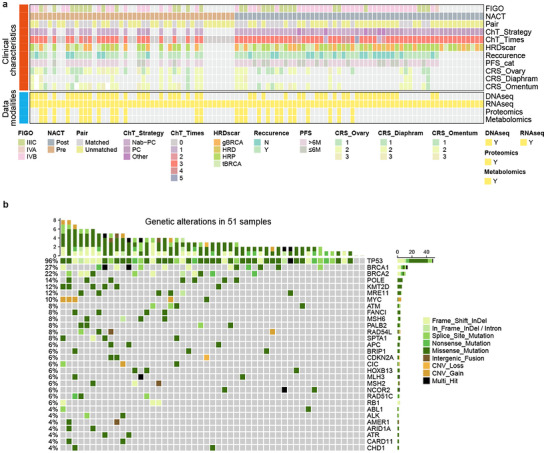
Overview of the study design and the genomic alteration. (a) The clinical characteristics including FIGO stage, treatment strategy, number of therapy cycles, BRCA status, CRS and prognosis, and data modalities including the sample number performed DNA, RNA, proteomics and metabolomics sequencing. (b) The genomic alterations of 51 patients. NACT, neoadjuvant chemotherapy.

We obtained 46 pre‐NACT and 56 post‐NACT tissue samples from 83 patients diagnosed with FIGO stage IIIC‐IV high‐grade serous ovarian cancer (HGSOC), including 19 matched pairs. Clinical characteristics are summarized in Table 1. All samples underwent RNA sequencing; among these, 51 had matched genomic profiling data routinely available from our institution, and 12 paired samples were subjected to proteomic and metabolomic analyses (Figure 1a). Patients were enrolled between October 2019 and August 2023.

**TABLE 1:** Clinical characteristics of the high‐grade serous ovarian cancer patients undergoing neoadjuvant chemotherapy.

Characteristics	DNA‐seq	RNA‐seq	Proteomics & Metabolomics
Samples	51	102	24
Patients	51	83	12
Paired	0	19	12
Median age at diagnosis, years (range)	58.1 (37‐69)	58.3 (37‐72)	57.3 (37‐67)
Median cycles of neoadjuvant chemotherapy (range)	3 (0‐5)	3 (0‐5)	3 (3‐4)
FIGO stage	—	—	—
IIIC	17 (33%)	18 (33%)	7 (59%)
IVA	2 (4%)	2 (3%)	1 (8%)
IVB	32 (63%)	35 (64%)	4 (33%)
NACT Strategy	—	—	—
Nab‐TP	32 (67%)	33 (54%)	12 (100%)
TP	11 (23%)	23 (38%)	0
Others	5 (10%)	5 (8%)	0
Tumor debulking status	—	—	—
Optimal	24 (57%)	25 (57%)	9 (75%)
Suboptimal	18 (43%)	19 (43%)	3 (25%)
Chemotherapy response score	—	—	—
1	18 (35%)	19 (36%)	3 (25%)
2	29 (57%)	30 (57%)	8 (67%)
3	4 (8%)	4 (7%)	1 (8%)
***Primary platinum response***	—	—	—
Sensitive	38 (75%)	40 (75%)	11 (12%)
Resistant	13 (25%)	13 (25%)	1 (8%)
BRCA	—	—	—
Wild	30 (59%)	35 (56%)	4 (33%)
Mutate	21 (41%)	28 (44%)	8 (67%)
Median Progression free survival, Months (range)	10.5(0‐27)	10.7 (0‐27)	14.3 (3‐22)

Within the post‐NACT cohort, 33 patients received neoadjuvant nab‐paclitaxel plus carboplatin as part of a clinical trial (ChiCTR1900026893), while 23 received conventional paclitaxel and carboplatin. The number of NACT cycles varied: 2 patients received 1 cycle, 3 patients received 2 cycles, 47 patients received 3 cycles, 3 patients received 4 cycles, and 1 patient received 5 cycles. Chemotherapy response was assessed using the Chemotherapy Response Score (CRS), evaluated independently by two expert gynecologic pathologists on omental, adnexal, or diaphragmatic specimens from 27 patients, following International Collaboration on Cancer Reporting criteria. The median follow‐up duration was 19 months (from interval debulking surgery to May 2024). Detailed clinicopathological features are provided in Figure 1a and Table 1.

TP53 variation was observed in 96% of patients. BRCA1 and BRCA2 were found exclusively mutated in 27% and 22% of patients. Genes with a mutation rate exceeding 10% also include POLE, KMT2D, MRE11, and MYC (Figure 1b).

### 2.2 Multi‐omics Profiling Uncovers Post‐NACT Alterations in Transcriptome, Proteome, and Metabolome

**FIGURE 2:**
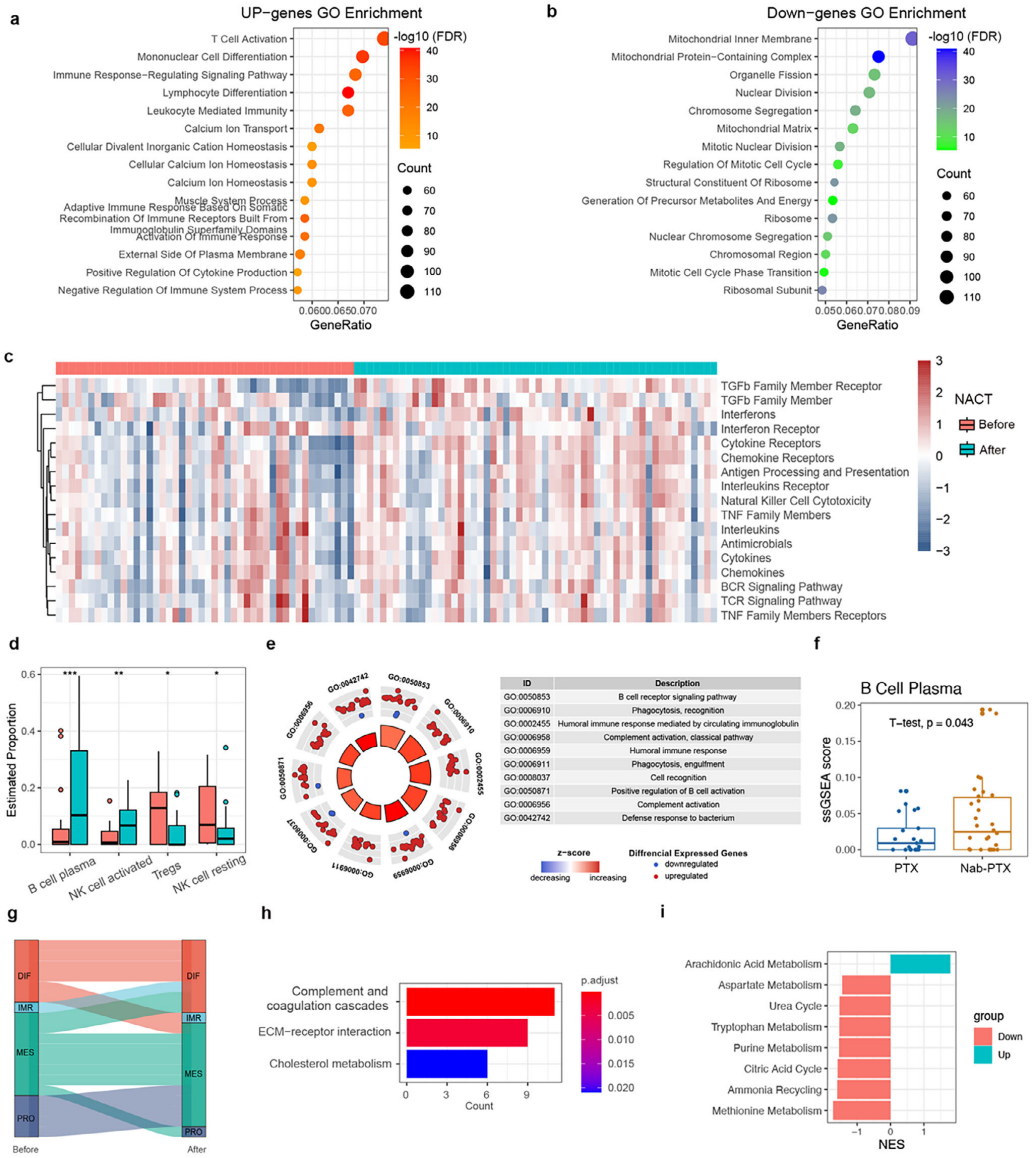
Transcriptomics, proteomics and metabolomics diversity following neoadjuvant chemotherapy. (a,b) The enrichment of upregulated genes (a) and downregulated genes (b) following neoadjuvant chemotherapy by transcriptomics (pre‐NACT, n = 46; post‐NACT, n = 56; unpaired cohorts, |log2fold change|≥1 and FDR<0.05). (c) Heatmap for immune‐related signatures following neoadjuvant chemotherapy by transcriptomics. (d) The diversity of infiltrated immune cells following neoadjuvant chemotherapy by transcriptomics. Data are presented as mean ± SEM; ^*^ *P*<0.05, ^**^ *P*<0.01, ^***^ *P*<0.005 by two‐tailed unpaired Student's *t*‐test. (e) Difference between post‐NACT samples treated with nab‐paclitaxel and carboplatin (n = 33) and those treated with paclitaxel and carboplatin (n = 23) by transcriptomics. (f) The difference in the infiltration of B cells between post‐NACT samples treated with nab‐paclitaxel and carboplatin and those treated with paclitaxel and carboplatin by transcriptomics. Data are presented as mean ± SEM; statistical significance was determined by two‐tailed unpaired Student's *t*‐test, with *p* values indicated in the figure. (g) The TCGA subtype following neoadjuvant chemotherapy by transcriptomics. (h, i) The diversity following neoadjuvant chemotherapy by proteomics (h) and metabolomics (i) (n = 12 paired cohorts; |log2 fold change| > 1.5 and FDR < 0.05).

Batch effects of RNA sequencing data from 46 pre‐NACT and 56 post‐NACT samples were corrected before analysis (Figure S1a), as confirmed by principal component analysis (PCA) colored by batch and treatment. It revealed distinct transcriptomic profiles that clearly distinguished treatment phases while maintaining overall aggregation (Figure S1b). We identified 1251 upregulated and 3069 downregulated genes following chemotherapy (Figure S1c). Upregulated differentially expressed genes (DEGs) were significantly enriched in immune‐related processes, including T cell activation, immune response‐regulating signaling pathways, and cellular calcium ion homeostasis. In contrast, downregulated DEGs were primarily associated with mitochondrial energy metabolism and cell proliferation, such as mitochondrial inner membrane organization, mitochondrial protein‐containing complexes, and nuclear division (Figure 2a,b). Consistent with previous reports [12, 13], post‐NACT tissues exhibited a more active immune microenvironment. Specifically, NACT significantly enhanced activity in TGF‐β family members and their receptors, B cell receptor (BCR) and T cell receptor (TCR) signaling pathways, interferon response, cytokine and chemokine activity, and antigen processing and presentation in paired samples. Among non‐paired tissues, TGF‐β receptor signaling and cytokine receptor pathways were also markedly activated after NACT (Figure 2c and Figure S1d,e). Furthermore, NACT altered the landscape of tumor‐infiltrating immune cells, increasing the proportions of plasma B cells and activated NK cells, while reducing regulatory T cells (Tregs) and resting NK cells in post‐NACT samples (Figure 2d and Figure S1f). These findings suggest that chemotherapy‐induced tumor cell death releases abundant tumor‐associated antigens, which in turn activate BCR‐mediated antigen presentation and promote B cell activation. This process ultimately enhances T cell‐mediated antitumor immunity through improved antigen presentation and pro‐inflammatory cytokine secretion.

We further compared the transcriptional profiles of post‐NACT samples treated with either nab‐paclitaxel plus carboplatin or paclitaxel plus carboplatin. Nab‐paclitaxel‐based treatment significantly enhanced B cell‐mediated immune activity, including the B cell receptor signaling pathway, circulating immunoglobulin‐mediated humoral immune response, and complement activation (Figure 2e,f). Consistent with this, compared to pre‐NACT tissues, nab‐paclitaxel exposure led to pronounced upregulation of complement activation, immunoglobulin receptor binding, and immunoglobulin complex formation (Figure S2a). We also evaluated the effect of chemotherapy cycle number on molecular profiles. Relative to 1–2 cycles, 3 cycles of NACT enhanced immune activation—including TNFα and interferon‐α/γ signaling—and suppressed proliferation‐related pathways such as KRAS signaling and E2F targets. In contrast, 4–5 cycles were associated with elevated resistance mechanisms, including MYC target activation and reactive oxygen species pathways, alongside reduced immune activity, supporting the molecular rationale for the clinical standard of 3 NACT cycles (Figure S2b,c). Notably, NACT altered TCGA molecular subtypes in 52.6% of patients, with an increased prevalence of Mesenchymal and Differentiated subtypes. All tumors originally classified as Proliferative transitioned to the Mesenchymal subtype (Figure 2g). These findings suggest that HGSOC subtypes undergo distinct evolutionary trajectories under chemotherapy pressure; whether these changes influence prognosis or other biological behaviors requires further investigation in larger cohorts.

Proteomic and metabolomic analyses were performed on paired samples. Batch correction was applied prior to differential analysis, and PCA confirmed effective removal of batch effects without disrupting biological clustering (Figure S3a). We identified 283 differentially expressed proteins (p < 0.05), including 97 downregulated and 183 upregulated proteins (Figure S3b). These proteins were significantly enriched in complement and coagulation cascades, ECM‐receptor interaction, and cholesterol metabolism pathways (Figure 2h). GSEA further revealed a reduction in DNA replication, spliceosome, and ribosome activity, alongside enhanced relaxin signaling, glycosaminoglycan binding, and exosome‐related functions after NACT (Figure S3c). NACT also induced significant metabolic alterations. We detected 134 differentially accumulated metabolites post‐treatment, implicating pathways such as DNA replication, pyrimidine and purine metabolism, and amino acid metabolism—including glutamate, beta‐alanine, and tyrosine metabolism (Figure S3d,e). In alignment with transcriptomic data, mitochondrial energy metabolism pathways were notably downregulated, particularly in aspartate metabolism and the citric acid cycle (Figure 2i). Integrated proteometabolomic analysis further confirmed pronounced activation of pantothenate and CoA biosynthesis, pyrimidine metabolism, and cysteine and methionine metabolism in post‐NACT samples (Figure S3f).

### 2.3 CD19^+^ B Cell Infiltration and Immune Activation Are Associated With Improved NACT Response

**FIGURE 3:**
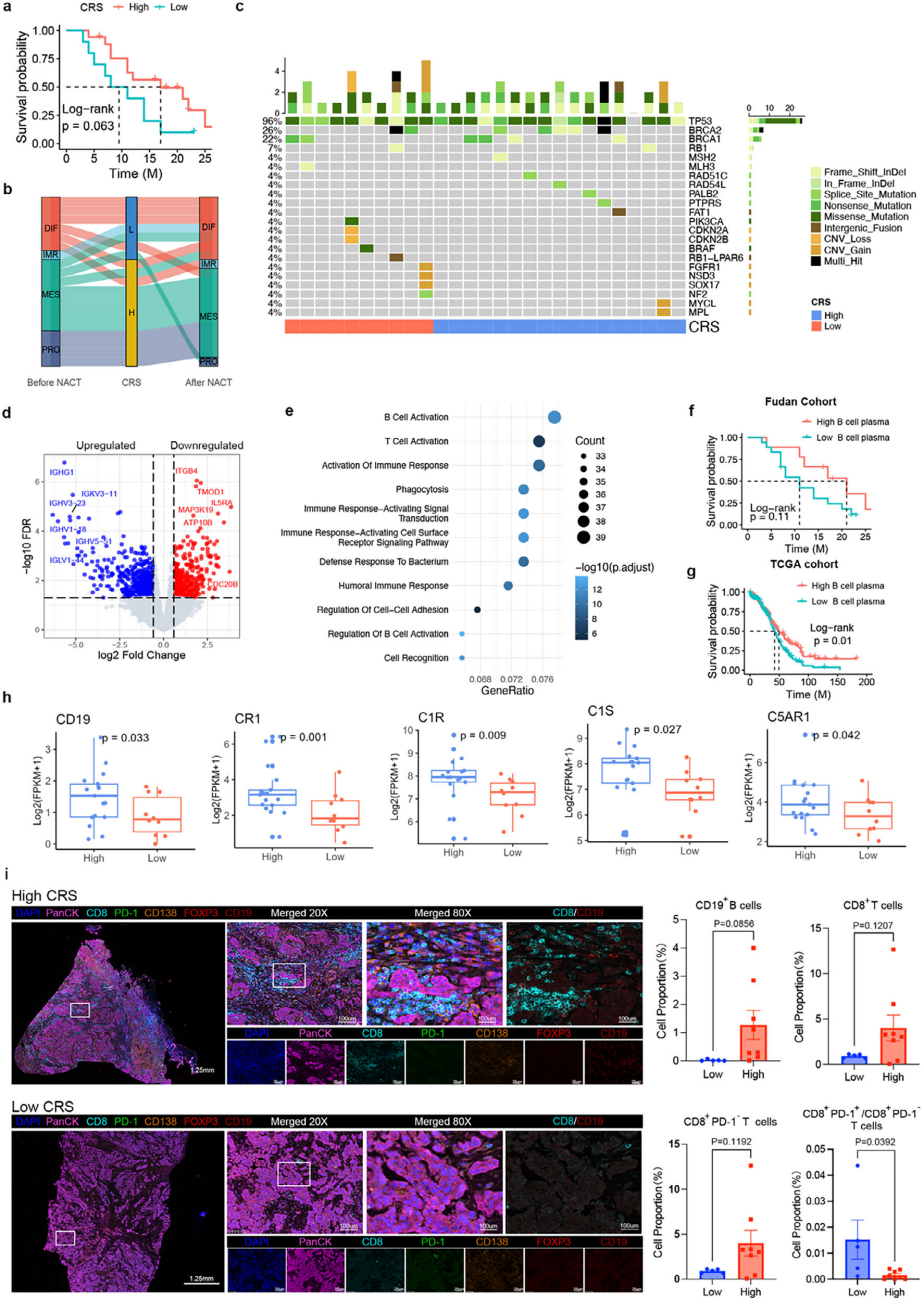
Molecular characteristics of high‐grade serous ovarian cancer patients withimproved response to neoadjuvant chemotherapy. (a) Progression‐free survival (PFS) of patients with high (n = 17) versus low (n = 10) chemotherapy response score (CRS). *p*‐value was calculated by the log‐rank test (median PFS: 17.1 months vs. 9.5 months, p = 0.063). (b) The TCGA subtype switching of patients with high and low CRS following NACT by transcriptomics in paired samples (n = 19). (c) The genomic alteration of patients with high (n = 17) and low (n = 10) CRS. (d) The differentially expressed genes of patients with low CRS (n = 10) compared to those with high CRS (n = 17). (e) The enriched immune pathway between samples with high (n = 17) and low (n = 10) CRS. (f) PFS of patients with high (n = 9) versus low (n = 18) B cell infiltration in our cohort. *p*‐value was calculated by the log‐rank test (median PFS: 21 months vs. 11 months, p = 0.11). (g) Overall survival (OS) of patients with high (n = 133) versus low (n = 173) B cell infiltration in the TCGA cohort. *p*‐value was calculated by the log‐rank test (median OS: 41.97 months vs. 49.47 months, p = 0.01). (h) Complement‐related signature enrichment scores in patients with high (n = 17) and low (n = 10) CRS based on transcriptomic data. Data are presented as mean ± SEM. Statistical significance was determined by two‐tailed unpaired Student's *t*‐test, with *p* values indicated in the figure. (i) Multiplex immunofluorescence staining of tumor samples from patients with high CRS (n = 5) and low CRS (n = 8). Representative images (left) and quantitative analysis (right) are shown. Visible structures include cytokeratin‐positive tumor cells (cyan), CD8^+^ T cells (light blue), PD‐1^+^ cells (green), CD138^+^ plasma cells (orange), FoxP3^+^ regulatory T cells (red), CD19^+^ B cells (dark red), and nuclei (blue). The scale bar is shown in the picture. Data are presented as mean ± SEM. Statistical significance was determined by two‐tailed unpaired Student's *t*‐test, with *p* values indicated in the figure. CRS, chemotherapy response score; PFS, progression‐free survival.

Chemotherapy Response Score (CRS) was evaluated in 27 patients with HGSOC. Although not statistically significant (p = 0.063), presumably due to limited sample size, patients with low CRS showed a non‐significant trend toward shorter progression‐free survival (Figure 3a). In both paired and unpaired tumor samples collected before and after NACT, chemotherapy increased the proportion of the *Differentiated* subtype among tumors with low CRS, while decreasing the proportion of the *Mesenchymal* subtype. Among tumors with high CRS, NACT slightly increased the proportion of the *Mesenchymal* subtype and reduced the proportion of the *Proliferative* subtype. (Figure 3b and Figure S4a). Patients with BRCA1/2 mutations showed a tendency toward higher CRS, though this correlation was not statistically significant (Figure 3c and Figure S4b). Similarly, mutations in homologous recombination repair‐related genes (e.g., *MSH2, RAD51C, RAD54L*) were associated with chemosensitivity, whereas alterations in proliferative genes (e.g., *PIK3CA, CDKN2A, CDKN2B*) correlated with low CRS (Figure 3c).

**FIGURE 4:**
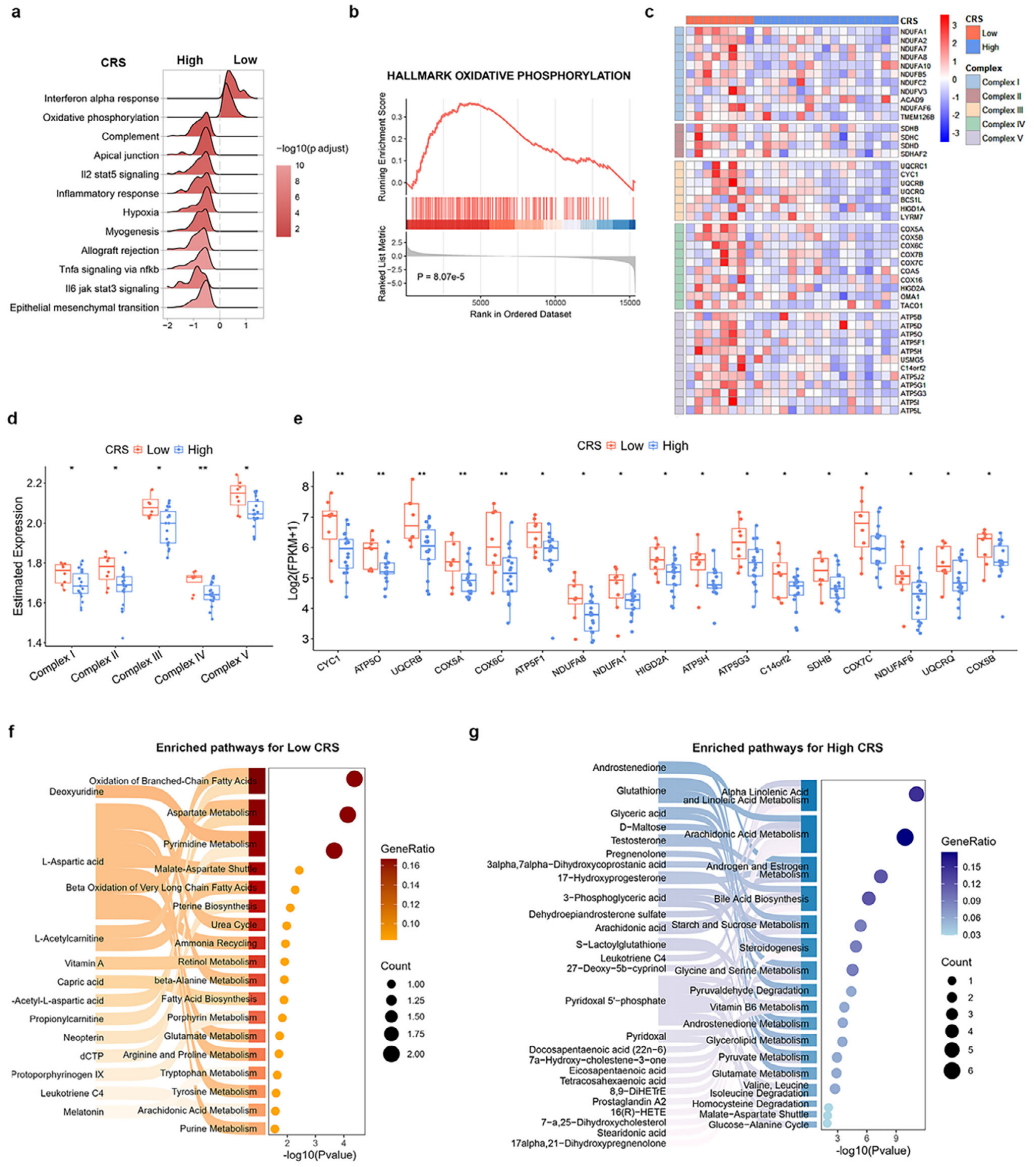
Patients with highly activated oxidative phosphorylation are correlated with poor response to neoadjuvant chemotherapy. (a) Gene set enrichment analysis (GSEA) of differentially expressed genes between pre‐treatment samples from patients with high CRS (n = 17) and low CRS (n = 10). Significantly enriched pathways were identified based on a normalized enrichment score (|NES| > 1) and a false discovery rate (FDR) q‐value <0.25. (b) The activation of oxidative phosphorylation in pre‐treatment samples with low CRS (n = 10). (c) Differentially expressed genes (DEGs) related to oxidative phosphorylation Complex I‐IV between pre‐treatment samples with high CRS (n = 17) and low CRS (n = 10). DEGs were filtered based on the thresholds of |log2FC| ≥ 1 and FDR < 0.05. (d) Overall expression score of oxidative phosphorylation Complex I–IV in pre‐treatment samples with high CRS (n = 17) and low CRS (n = 10). Data are presented as mean ± SEM. ^*^ *P*<0.05, ^**^ *P*<0.01 by two‐tailed unpaired Student's *t*‐test. (e) Volcano plot highlighting statistically significant differentially expressed oxidative phosphorylation‐related genes between high CRS (n = 17) and low CRS (n = 10) pre‐treatment samples (|log2FC| ≥1 and FDR <0.05). Data are presented as mean ± SEM, ^*^ *P*<0.05, ^**^ *P*<0.01 by two‐tailed unpaired Student's *t*‐test. (f,g) Integrated metabolomics and transcriptomics analysis revealing enriched metabolites and associated pathways in CRS‐low (n = 10, f) and CRS‐high (n = 17, g) pre‐treatment samples. Dot size represents pathway impact score; dot color indicates significance level (−log_10_(*p*‐value)).

Transcriptomic profiling identified 859 upregulated and 551 downregulated genes in high‐CRS tumors, indicating distinct molecular phenotypes (Figure S4c). Low‐CRS tumors overexpressed driver genes such as *ITGB4, MAP3K19*, and *ATP10B*, but exhibited reduced expression of B cell‐related immunoglobulin genes (e.g., *IGHG1, IGKV3‐11*; Figure 3d). Gene set enrichment analysis highlighted significant immune activation in high‐CRS tumors, including B and T cell activation pathways (Figures 3e and 4a). Transcriptional comparison between platinum‐sensitive and resistant tissues reinforced these findings: platinum‐sensitive samples were enriched in B cell activation, immunoglobulin complexes, and BCR signaling, while resistant samples showed elevated mitochondrial electron transport chain activity (Figure S5). High B cell infiltration—particularly plasma B cells—was associated with improved PFS in both our cohort and TCGA data (Figure 3f,g). High‐CRS tumors exhibited pronounced B cell‐mediated immune activity, with elevated expression of complement components (CR1, C1R, C1S, C5AR1), chemokines (CCL21, CCL5, CXCL9), cytokines, and immunoglobulins (Figure 3h and Figure S6). Multiplex immunohistochemistry confirmed an immunoactive microenvironment in high‐CRS tumors, showing increased infiltration of CD19^+^ B cells, CD8^+^ T cells, and CD8^+^PD‐1^−^ T cells, though not all reached significance. The ratio of CD8^+^PD‐1^+^/CD8^+^PD‐1^−^ T cells was significantly higher in low‐CRS tumors (Figure 3i). Notably, the average CD19^+^ B cell infiltration in high‐CRS tumors was significantly higher than that in low‐CRS tumors (1.28% vs. 0.03%). In five patients with CD19^+^ B cell infiltration ≥1%, the R0 resection rate was 100%, compared to 60% in those with <1% infiltration, suggesting CD19^+^ B cell abundance may identify patients most likely to benefit from NACT. Furthermore, multivariable Cox regression analysis, adjusting for potential confounders such as chemotherapy regimen, treatment cycles, FIGO stage, BRCA status, confirmed that higher CD19^+^ B cell infiltration remained an independent predictor of favorable survival in ovarian cancer patients (Table S1). In summary, CD19^+^ B cell infiltration is associated with improved NACT response and higher R0 resection rates in advanced HGSOCs.

### 2.4 Elevated OXPHOS Activity Defines NACT‐Resistant Ovarian Tumors

Next, we sought to dissect the molecular hallmarks underlying the NACT‐resistant phenotype associated with elevated OXPHOS activity in HGSOC. Transcriptomic profiling revealed that low CRS tumors were associated with robust upregulation of oxidative phosphorylation (OXPHOS) pathways (Figure 4a,b). Specifically, nearly all major genes encoding subunits of the mitochondrial electron transport chain (ETC) complexes I–IV were significantly elevated in low CRS compared to high CRS tumors (Figure 4c,d). Among these, CYC1, ATP5O, UQCRB, COX5A and COX6C were markedly upregulated (p < 0.01, Figure 4e). Metabolomic analysis further identified 134 differentially enriched metabolites (FDR < 0.05) in low CRS tumors. Most accumulated metabolites were implicated in the Tricarboxylic Acid Cycle (TCA cycle)—including the malate‐aspartate shuttle, branched‐chain fatty acid oxidation, and amino acid metabolism supporting ETC and mitochondrial respiration. In contrast, high CRS tumors were characterized by enrichment of polyunsaturated fatty acids (e.g., alpha‐linolenic and arachidonic acid metabolism), as well as estrogen, pyruvate, and pyrimidine metabolic pathways (Figure 4f,g). Together, these multi‐omics findings indicate that heightened OXPHOS activity and the associated rewiring of mitochondrial energy metabolism constitute defining hallmarks of NACT‐resistant HGSOC, underscoring OXPHOS as a pivotal metabolic mediator of chemoresistance.

### 2.5 Enhanced Mitochondrial OXPHOS Activity Underlies Carboplatin Resistance in High‐grade Serous Ovarian Cancer Cells and Patient‐Derived Organoids

**FIGURE 5:**
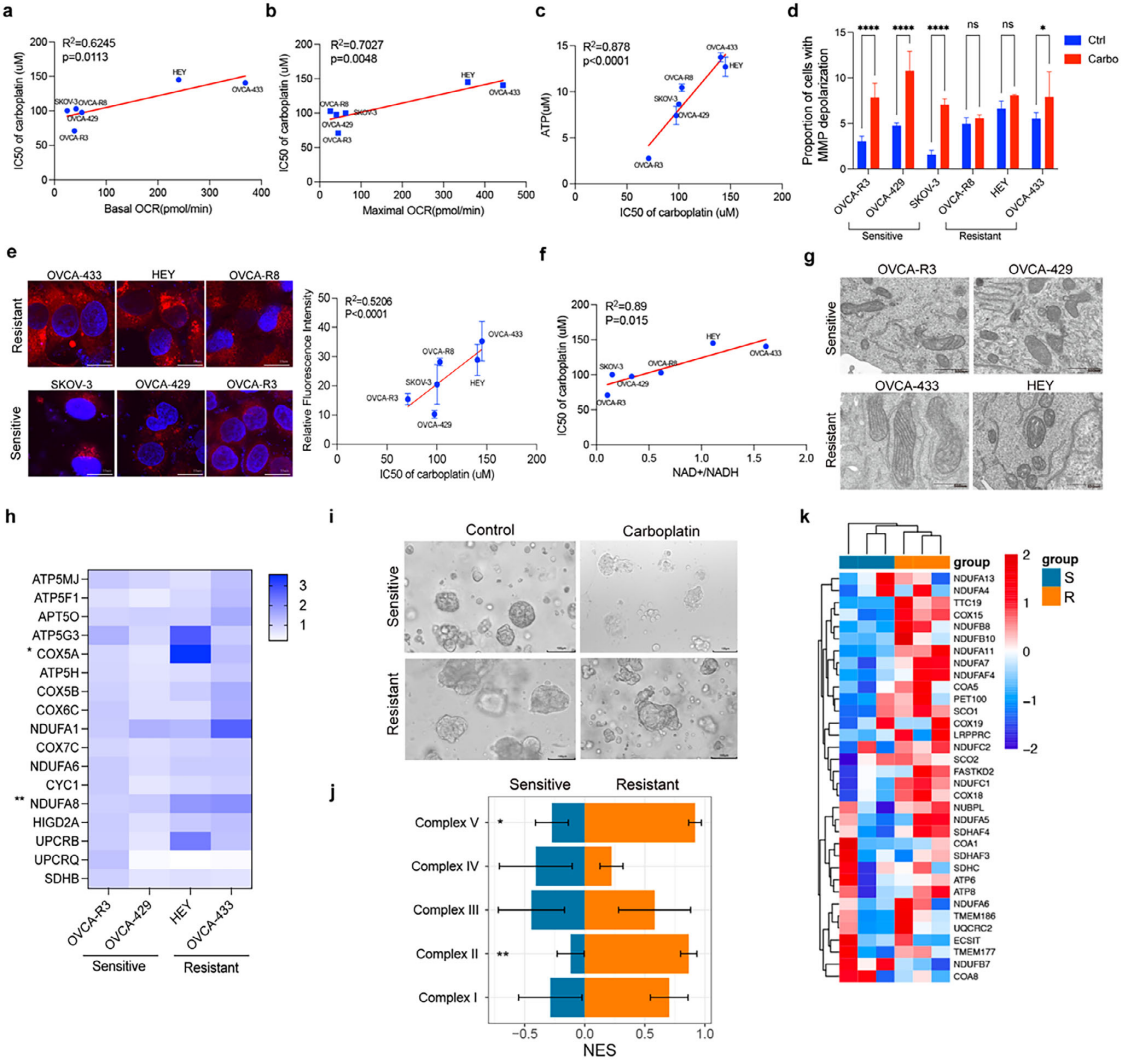
Carboplatin‐resistant ovarian cancer cells exhibit enhanced oxidative phosphorylation activity. (a–c) Correlation between carboplatin IC_50_ values and basal oxygen consumption rate (OCR) (a), maximal OCR (b), and ATP production (c, n = 3 per cell line) in six ovarian cancer cell lines. Spearman correlation coefficients (r) and *p* values are indicated; data are presented as mean ± SD. (d) The proportion of cells with mitochondrial membrane potential (MMP) depolarization in carboplatin‐sensitive and ‐resistant ovarian cancer cells following carboplatin treatment (n = 3 per group). Data are presented as mean ± SD. ^*^ *P*<0.05, ^****^ *P*<0.001, ns, not significant by two‐tailed unpaired Student's *t*‐test. (e) Representative images (left) and quantitative correlation analysis (right) between MitoTracker fluorescence intensity and carboplatin IC_50_ values across 6 ovarian cancer cell lines. Scale bar = 10 µm. For each cell line, fluorescence intensity data are presented as mean ± SD (n = 4). The two‐tailed Spearman correlation coefficient (R) and *p*‐value calculated from the cell line means are indicated directly in the figure. (f) Correlation between the calculated NAD^+^/NADH ratio and carboplatin IC_50_ values across 6 ovarian cancer cell lines. The NAD^+^/NADH ratio for each cell line was determined from the respective measurements of NAD^+^ and NADH (n = 3). The two‐tailed Spearman correlation coefficient (R) and *p*‐value are indicated directly in the figure. (g) Representative transmission electron microscopy (TEM) images of carboplatin‐sensitive and resistant cell lines. Scale bars = 0.5 µm. (h) Relative expression levels of 17 electron transport chain (ETC) genes (from Figure 4e) in carboplatin‐sensitive and ‐resistant ovarian cancer cells (n = 4 per cell line per gene). For each gene, statistical significance between the sensitive and resistant groups was determined by two‐tailed unpaired Student's *t*‐test; ^*^ *P*<0.05, ^**^ *P*<0.01. (i) The representative image of sensitive and resistant PDOs on carboplatin, Scale bar = 100 µm. (j) Composite expression score of ETC Complex I–IV genes in PDOs with high IC_50_ (n = 3) and low IC_50_ (n = 3) to carboplatin. Data are presented as mean ± SD. ^*^ *P*<0.05, ^**^ *p* < 0.01 by two‐tailed unpaired Student's *t*‐test. (k) Heatmap showing differentially expressed ETC genes between PDOs with high and low carboplatin IC_50_ values. Color scale represents row‐scaled expression levels. PDOs, patient‐derived organoids; ETC, electron transport chain; OCR, oxygen consumption rate; MMP, mitochondrial membrane potential; TEM, transmission electron microscopy; SD, standard deviation.

To elucidate the relationship between platinum resistance and OXPHOS activity, we evaluated six commonly used ovarian cancer cell lines (HEY, OVCA‐429, OVCA‐R3, OVCA‐433, SKOV3, OVCA‐R8) and patient‐derived organoids (PDOs). Carboplatin sensitivity, assessed by IC_50_, varied widely across cell lines (Figure S7a). Mitochondrial respiratory function, reflected by basal and maximal oxygen consumption rates (OCR), was strongly correlated with carboplatin resistance (basal OCR: R = 0.6245, p = 0.0113; maximal OCR: R = 0.7027, p < 0.001; Figure S7b and Figure 5a,b). In contrast, extracellular acidification rate (ECAR) did not differ significantly between sensitive and resistant models (Figure S7c). A particularly strong correlation was observed between IC_50_ and mitochondrial ATP production (R = 0.878, p < 0.001; Figure 5c). Given the central role of mitochondrial membrane potential (MMP, ΔΨm) in sustaining OXPHOS and ATP generation, we compared MMP across models and observed elevated basal MMP in carboplatin‐resistant cells. Carboplatin treatment induced pronounced MMP collapse in sensitive cells but had minimal effects in resistant lines, suggesting mitochondrial involvement in carboplatin‐induced apoptosis (Figure 5d). Consistent with this, MitoTracker Red CMXRos intensity—reflecting functional mitochondrial mass—was strongly associated with carboplatin resistance, that platinum resistant cell lines had higher fluorescence intensity than that in platinum‐sensitive groups (Figure 5e). Resistant cells also exhibited a persistent pro‐oxidant state, marked by glutathione (GSH) depletion and an elevated NAD^+^/NADH ratio (Figure S7d and Figure 5f). ROS levels, indicative of OXPHOS activity, were also positively correlated with IC_50_ (Figure S7e). Electron microscopy revealed more abundant cristae and better‐preserved mitochondrial ultrastructure in resistant lines (OVCA‐433, HEY) compared to sensitive ones (OVCA‐R3, OVCA‐429; Figure 5g). Expression of key electron transport chain (ETC) genes was similarly upregulated in resistant cells (Figure 5h), collectively establishing enhanced ETC/OXPHOS activity as a hallmark of carboplatin‐resistant ovarian cancer.

These findings were further validated in six HGSOC patient‐derived organoids (PDOs) stratified by carboplatin sensitivity based on IC_50_ values (Figure 5i and Figure S8). Resistant PDOs exhibited elevated expression scores for Complex I, II and V (Figure 5j), with Complex I showing a consistent upward trend—though not statistically significant—along with higher transcript levels of mitochondrial respiration‐related genes, such as NDUFA7, NDUFB8, NDUFA11 (Figure 5k). Together, these results position high OXPHOS activity as a key candidate predictive biomarker of chemotherapy resistance in HGSOC.

### 2.6 Disruption of Complex I–Dependent Mitochondrial Homeostasis Reverses Platinum Resistance Through Mitophagy‐Associated Stress

**FIGURE 6:**
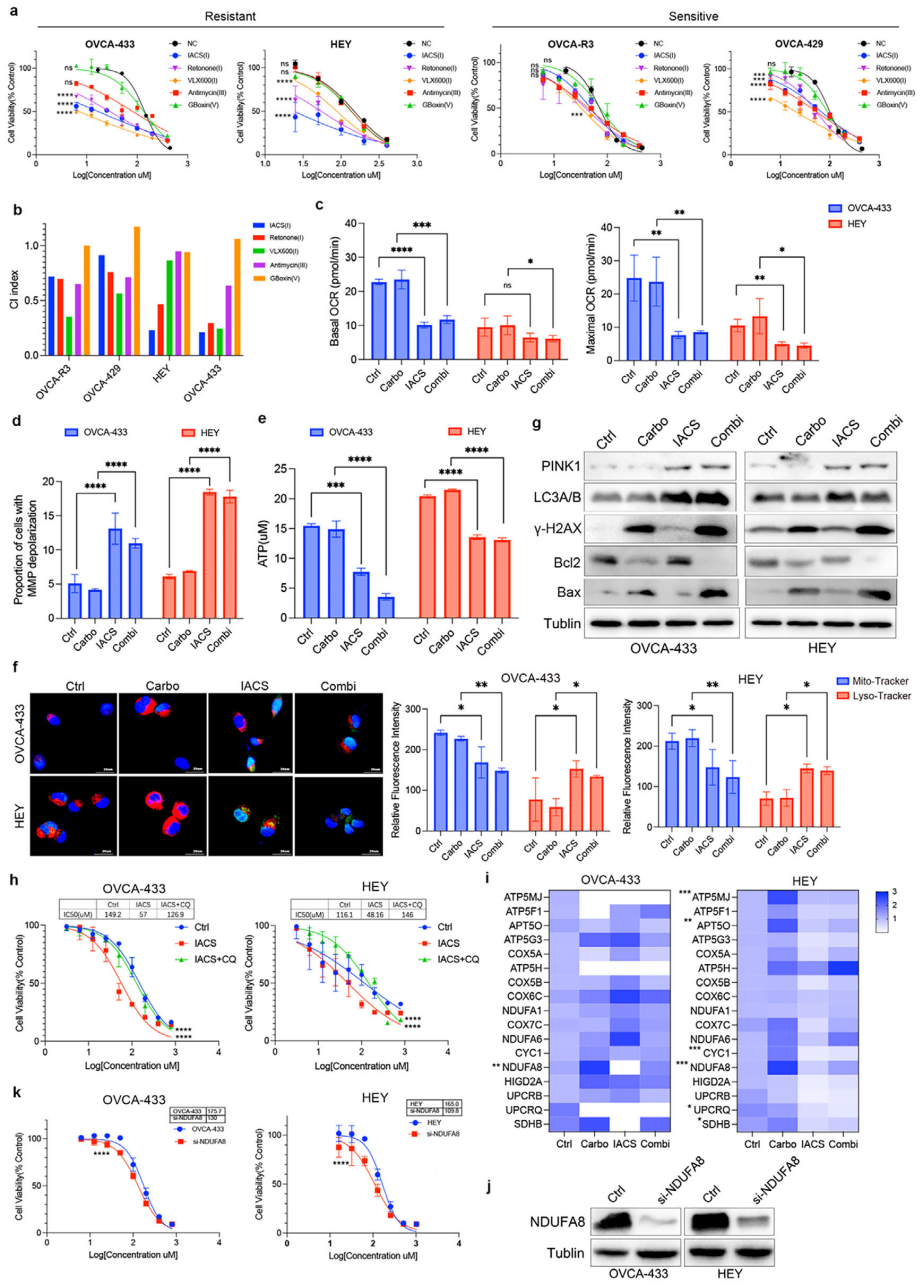
Disruption of Complex I–dependent mitochondrial homeostasis reverses platinum resistance through mitophagy‐associated stress. (a) Cell viability of carboplatin‐resistant (left) and ‐sensitive (right) ovarian cancer cells treated with increasing concentrations of Complex inhibitors: Complex I inhibitors (IACS‐010759, VLX600, rotenone), Complex III inhibitor (antimycin), and Complex V inhibitor (oligomycin A). Data are presented as mean ± SD (n = 3 per group), ^***^ *p* < 0.005, ^****^ *p* < 0.001, ns, not significant by two‐way ANOVA followed by Tukey's post hoc test. (b) Combination index (CI) values of Complex inhibitors in carboplatin‐resistant and ‐sensitive ovarian cancer cells when combined with carboplatin. CI < 1 indicates a synergistic effect. (c–e) Effects of IACS‐010759 treatment on basal and maximal OCR (c), the proportion of cells with mitochondrial membrane potential (MMP) depolarization (d), and intracellular ATP levels (e) in carboplatin‐resistant OVCA‐433 and HEY cells. Data are presented as mean ± SD (n = 3 per group). ^*^ *p* < 0.05, ^**^ *p* < 0.01, ^***^ *p* < 0.005, ^****^ *p* < 0.001 and ns, not significant by two‐tailed unpaired Student's *t*‐test. (f) Representative confocal immunofluorescence images (left) and quantitative analysis (right) of carboplatin‐resistant ovarian cancer cells treated with IACS‐010759. Mitochondria were labeled with MitoTracker (green), lysosomes with LysoTracker (red). Scale bar = 100 µm. Data are presented as mean ± SD (n = 3 per group). ^*^ *p* < 0.05, ^**^ *p* < 0.01 by two‐tailed unpaired Student's *t*‐test. (g) Immunoblot analysis of PINK1, LC3‐II, γH2AX and apoptosis‐related Bcl2 and Bax protein levels following Complex I inhibition by IACS‐010759 in carboplatin‐resistant ovarian cancer cells. Representative images from three independent experiments are shown. (h) Synergistic cytotoxic effect of IACS‐010759 and carboplatin in the presence or absence of the autophagy inhibitor chloroquine (CQ). Cell viability was assessed by CCK‐8. Data are presented as mean ± SD (n = 3 per group), ^****^ *p* < 0.001 by two‐way ANOVA followed by Tukey's post hoc test. (i) Relative mRNA expression levels of 17 electron transport chain (ETC) genes in carboplatin‐resistant OVCA‐433 and HEY cells following IACS‐010759 treatment (n = 4 per gene). For each gene, statistical significance between the sensitive and resistant groups was determined by two‐tailed unpaired Student's *t*‐test; ^*^ *p* < 0.05, ^**^ *p* < 0.01, ^***^ *p* < 0.005. (j) Representative Western blot images showing NDUFA8 protein levels in OVCA‐433 and HEY cells transfected with control siRNA(Ctrl) or siRNA targeting NDUFA8 (si‐NDUFA8). (k) IC_50_ values of carboplatin in OVCA‐433 and HEY cells following siRNA‐mediated knockdown of NDUFA8. Data are presented as mean ± SD (n = 3 per group), ^****^ *p* < 0.001 by two‐way ANOVA followed by Tukey's post hoc test. CI, combination index; OCR, oxygen consumption rate; MMP, mitochondrial membrane potential; ETC, electron transport chain; CQ, chloroquine; SD, standard deviation.

To identify the specific mitochondrial electron transport chain (ETC) complex critical to platinum resistance in HGSOC, we evaluated clinically validated inhibitors targeting Complex I (IACS‐010759, VLX600, Rotenone), III (Antimycin A), and V (Oligomycin A) in both platinum‐resistant (OVCA‐433, HEY) and sensitive (OVCA‐R3, OVCA‐429) cell lines. Pharmacological inhibition of Complex I most potently synergized with carboplatin in resistant cells, as demonstrated by significantly lower combination index (CI) values for IACS‐010759 and rotenone compared to inhibitors of Complex III or V. In contrast, no significant synergy was observed in platinum‐sensitive lines (Figure 6a,b), suggesting a selective dependency of resistant cells on Complex I activity.

Given that IACS‐010759 selectively inhibits mitochondrial Complex I, we systematically assessed mitochondrial integrity and metabolic homeostasis in carboplatin‐resistant cells following treatment. Oxygen consumption rate (OCR) analysis revealed marked suppression of both basal and maximal respiration, indicating impaired oxidative phosphorylation (Figure 6c and Figure S9a). Consistently, IACS‐010759 disrupted NAD^+^ homeostasis, reflected by alterations in the NAD^+^/NADH ratio, further supporting defective electron transport chain activity (Figure S9b). In line with compromised respiratory function, the proportion of cells with MMP depolarization, indicating mitochondrial damage, was significantly elevated in the combinational group compared to that of the carboplatin group (Figure 6d). Transmission electron microscopy revealed swollen mitochondria with disrupted cristae architecture, confirming structural mitochondrial damage (Figure S9c). Concomitantly, intracellular ATP levels were moderately but significantly decreased, accompanied by increased ROS accumulation and reduced GSH levels, indicating redox imbalance (Figure 6e and Figure S9d,e). Together, these findings demonstrate that Complex I inhibition induces coordinated mitochondrial respiratory suppression, membrane depolarization, structural disruption, and oxidative stress.

Transcriptomic profiling further revealed downregulation of oxidative phosphorylation gene signatures and activation of stress‐responsive pathways following IACS‐010759 treatment (Figure S9f). In line with mitochondrial depolarization and oxidative stress, we observed stabilization of PINK1 and accumulation of LC3‐II, accompanied by increased lysosomal but decreased mitochondrial colocalization, indicating engagement of mitophagic responses (Figure 6f,g). This metabolic shift triggered profound genomic instability, evidenced by elevated γH2AX levels and increased DNA “tail moments” in Comet assays (Figure 6g and Figure S9g). The resulting DNA lesions induced a robust DNA damage response (DDR), characterized by the upregulation of ATR and RAD51 (Figure S9h). Ultimately, the inability to resolve this replication stress, coupled with a bioenergetic crisis, forced a terminal shift in the apoptotic threshold—marked by the downregulation of Bcl‐2 and the concurrent activation of Bax (Figure 6g). To further delineate the molecular underpinnings of this sensitization, we performed functional rescue experiments using the autophagy inhibitor Chloroquine (CQ). This showed that CQ significantly attenuated the synergistic cytotoxicity between the Complex I inhibitor and carboplatin (Figure 6h). At the molecular level, CQ treatment partially rescued the DNA tailing and γH2AX levels, while effectively reversing the pro‐apoptotic transition—characterized by the suppression of Bax elevation and the partial recovery of Bcl‐2 expression (Figure S9g,i). In summary, Complex I inhibition triggers an excessive mitophagic response, which potentially creates a bioenergetic deficit that impairs ATP‐dependent DNA repair. This unresolved genomic stress, sensed by the ATR/RAD51 pathway, likely shifts the Bax/Bcl‐2 balance to favor apoptosis, identifying the “Complex I–PINK1–ATR/RAD51–Bax/Bcl‐2” axis as a key mechanism for carboplatin resensitization

To further delineate the molecular determinants underlying Complex I–dependent vulnerability, we examined the expression of representative ETC subunits following IACS‐010759 treatment. Among these, NDUFA8 exhibited the most pronounced downregulation, implicating it as a potential structural node mediating the observed bioenergetic disruption (Figure 6i). To genetically interrogate this possibility, we performed siRNA‐mediated knockdown of key subunits across mitochondrial Complexes I–V. Notably, silencing of NDUFA8 significantly restored carboplatin sensitivity in resistant OVCA‐433 and HEY cells, as evidenced by a marked reduction in IC_50_ values. In contrast, knockdown of subunits from Complex II (SDHB), Complex III (COX6C, CYC1), or Complex V (ATP5O) did not significantly alter carboplatin responsiveness, indicating a selective dependency on Complex I integrity (Figure 6j and Figure S10a,b). Mechanistically, NDUFA8 depletion recapitulated the mitochondrial defects induced by pharmacologic Complex I inhibition. Transcriptomic profiling revealed suppression of oxidative phosphorylation‐related gene signatures and enrichment of metabolic stress pathways. Interestingly, transcriptomic analysis further revealed enrichment of immune‐related pathways upon NDUFA8 depletion, particularly gene signatures associated with B cell‐mediated immune responses. This observation is consistent with our earlier findings linking B cell‐associated immune activation to improved chemotherapy responsiveness, suggesting that disruption of mitochondrial bioenergetic stability may also reshape the tumor‐immune transcriptional landscape toward a more chemosensitive state. (Figure S10c–e). Consistently, functional assays demonstrated reduced mitochondrial membrane potential, increased mitochondrial ROS accumulation, and decreased intracellular ATP levels, collectively indicating impaired respiratory capacity and redox imbalance (Figure S10f–h). These alterations occurred in the absence of overt baseline cytotoxicity, suggesting that disruption of NDUFA8 primarily compromises mitochondrial functional robustness rather than directly inducing cell death.

**FIGURE 7:**
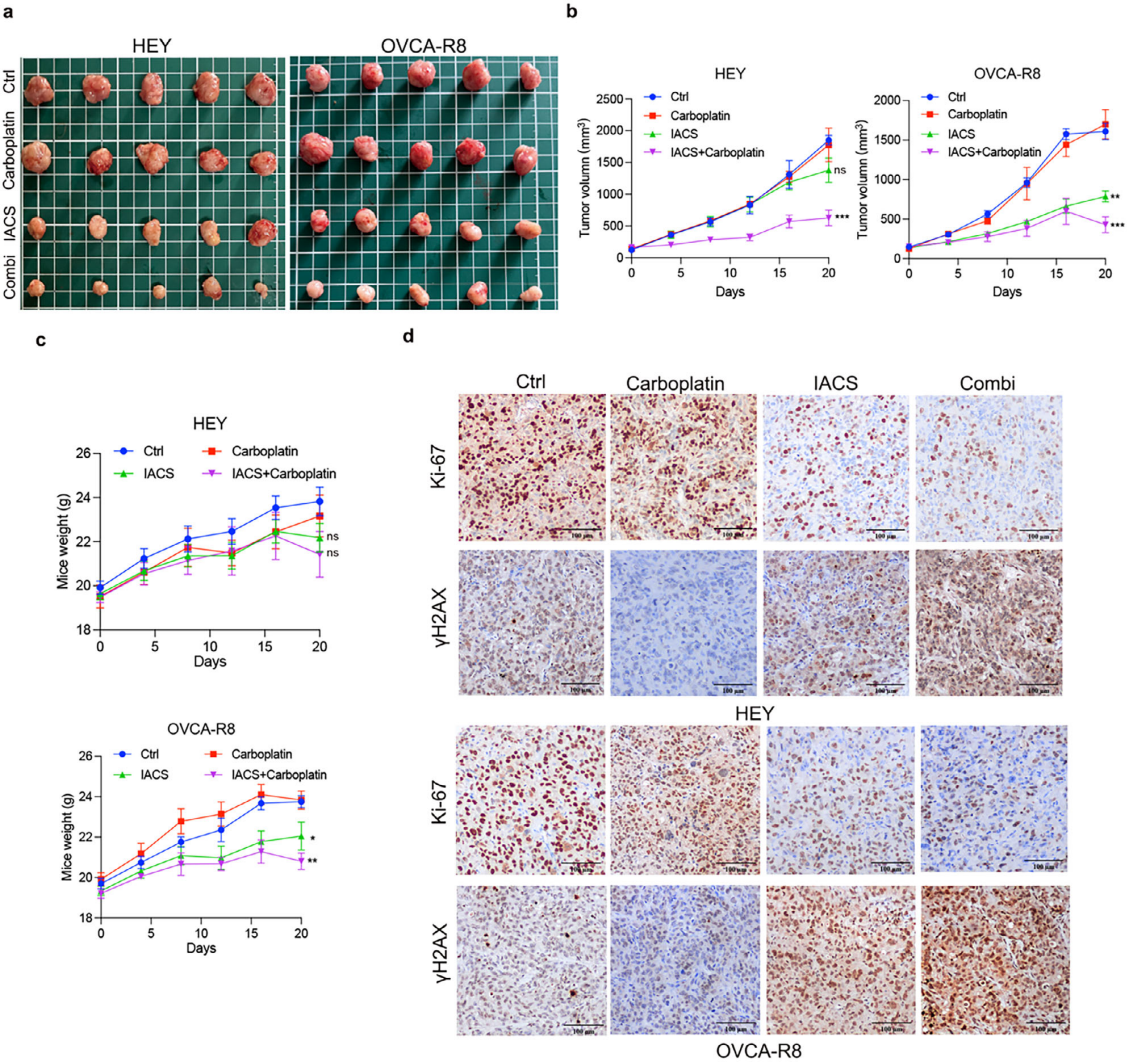
Mitochondrial Complex I inhibitor increases the sensitivity to carboplatin in platinum‐resistant ovarian cancer xenografts. Subcutaneous xenograft models were established using platinum‐resistant OVCA‐R8 and HEY cells. Treatment was initiated when tumor volumes reached approximately 1500 mm^3^. Mice were divided into four groups: control, carboplatin, IACS‐010759, and the combination. (a) Representative images of resected tumors from each treatment group (n = 5 mice per group). (b) Tumor growth curves over time for each treatment group. Data are presented as mean ± SEM. ^***^ *p* < 0.005 for combination vs. carboplatin monotherapy; ^**^ *p* < 0.01 and ns, not significant for IACS vs. Ctrl by two‐way ANOVA followed by Tukey's post hoc test. (c) Body weight changes of mice during the intervention period. Data are presented as mean ± SEM. ^*^ *p* < 0.05, ^**^ *p* < 0.01, and ns, not significant for combination and IACS vs. Ctrl by two‐way ANOVA followed by Tukey's post hoc test. (d) Representative immunohistochemical staining images (left) and quantitative analysis (right) of Ki67 (proliferation marker) and γH2AX (DNA damage marker) in tumor tissues from each treatment group. Scale bars = 100 µm. SEM, standard error of the mean.

The efficacy of IACS‐010759 in restoring platinum sensitivity was further validated in platinum‐resistant cell line‐derived xenograft models. Carboplatin monotherapy showed minimal inhibitory effect on tumor growth in both HEY and OVCA‐R8 xenografts. In contrast, IACS‐010759 single‐agent treatment induced tumor shrinkage, an effect that reached statistical significance in the OVCA‐R8 model. Notably, the combination of IACS‐010759 and carboplatin resulted in significant tumor suppression in both models (Figure 7a,b). Regarding treatment‐related toxicity, body weight measurements revealed that neither IACS‐010759 alone nor in combination with carboplatin significantly affected mouse body weight in the HEY model. However, both regimens caused significant weight reduction in OVCA‐R8 models (Figure 7c), indicating model‐specific adverse effects. Compared to the control group, carboplatin monotherapy resulted in minimal changes in Ki67 and γH2AX expression. In contrast, IACS‐010759 monotherapy led to significantly reduced Ki67 and increased γH2AX levels, with the most pronounced effects observed in the combination group (Figure 7d). These results demonstrate that inhibition of Complex I can effectively sensitize ovarian cancer cells to carboplatin.

Collectively, these findings identify NDUFA8 as a key determinant of Complex I‐mediated mitochondrial stability. Together with pharmacologic inhibition data, our results indicate that platinum‐resistant ovarian cancer depends on this axis for survival, and its disruption enhances oxidative stress and restores platinum sensitivity.

### 2.7 CD19^+^ B Cells and NDUFA8 Are Associated With Response to NACT in High‐grade Serous Ovarian Cancer

**FIGURE 8:**
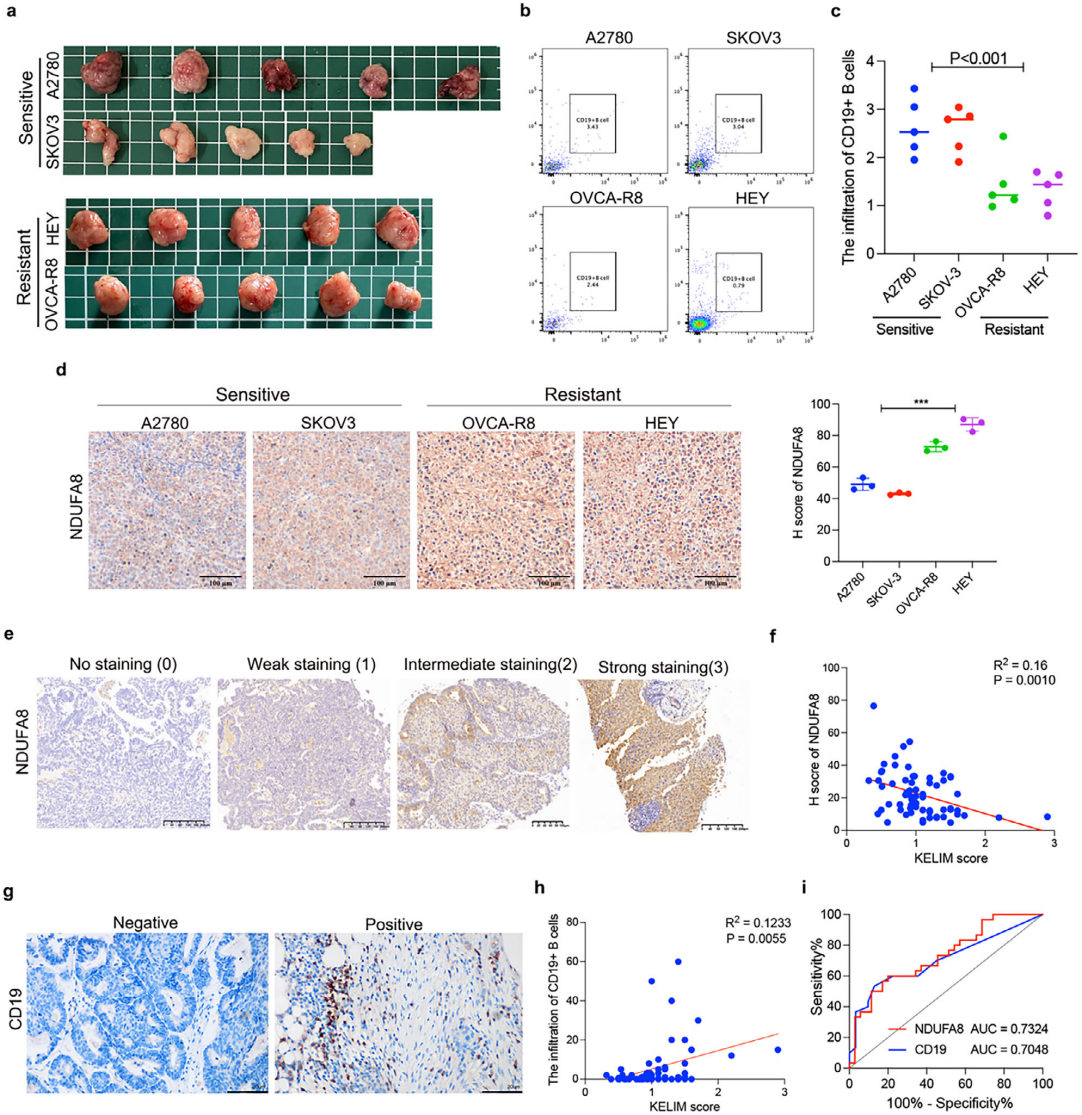
Infiltration of CD19^+^ B cells and the expression of NDUFA8 correlate with the response to neoadjuvant chemotherapy in high‐grade serous ovarian cancer. (a) Subcutaneous xenograft tumors derived from platinum‐sensitive (A2780 and SKOV‐3) and platinum‐resistant (OVCA‐R8, HEY) cell lines (n = 5 mice per group), excised when reaching visually comparable sizes. The representative image of the tumors. (b) Representative flow cytometry plots showing the number of tumor‐infiltrating B cells (CD19^+^) in platinum‐sensitive and resistant groups (n = 5 mice per group). (c) Quantitative analysis of CD19^+^ B cell infiltration levels in platinum‐sensitive versus platinum‐resistant xenografts. Each dot represents an individual mouse (n = 5 per group). Statistical significance was determined by two‐tailed unpaired Student's *t*‐test, with *p* values indicated. (d) Representative immunohistochemical (IHC) staining images (left) and quantitative analysis (right) of NDUFA8 expression in platinum‐sensitive and platinum‐resistant xenograft tumors. Scale bar = 100 µm. Each dot represents an individual mouse (n = 3 per group). ^***^ *p* < 0.005 by two‐tailed unpaired Student's *t*‐test. (e) Representative IHC images of NDUFA8 expression in pre‐neoadjuvant chemotherapy biopsy samples from patients with high‐grade serous ovarian cancer. Scale bar = 100 µm. (f) Correlation analysis between NDUFA8 expression and KELIM index in the validation cohort (n = 65). The two‐tailed Spearman correlation coefficient (r) and *p*‐value are indicated in the figure. (g) Representative images of CD19^+^ B cell infiltration in pre‐treatment biopsy samples, captured under identical magnification (40×) and uniform exposure settings. Scale bar = 20 µm. (h) Pearson correlation analysis between CD19^+^ B cell infiltration levels and KELIM score in the validation cohort (n = 61). The two‐tailed Spearman correlation coefficient (r) and *p*‐value are indicated in the figure. (i) Receiver operating characteristic (ROC) curves evaluating the predictive value of CD19^+^ B cell infiltration (blue, n = 61), NDUFA8 expression (red, n = 65) in the validation cohort. The area under the curve (AUC) values are indicated directly in the figure. SEM, standard error of the mean; IHC, immunohistochemistry; KELIM, CA‐125 elimination rate constant K (KELIM) index; ROC, receiver operating characteristic; AUC, area under the curve.

To further evaluate the relevance of CD19^+^ B cells and NDUFA8 as candidate predictors of chemotherapy response in ovarian cancer, we developed xenograft models representing platinum‐sensitive and platinum‐resistant phenotypes. Due to difficulties in reliably generating tumors from the initially planned platinum‐sensitive lines OVCA‐429 and OVCA‐R3, we utilized A2780 (IC_50_ = 70 µM; sensitivity comparable to OVCA‐R3) as a surrogate platinum‐sensitive model. Tumors were collected when reaching visually comparable volumes across groups (Figure 8a). Flow cytometry revealed significantly higher CD19^+^ B cell infiltration in platinum‐sensitive tumors than in resistant ones (3.3% vs. 1.9%, *p* < 0.001; Figure 8b,c). In parallel, immunohistochemical analysis showed markedly elevated NDUFA8 expression in resistant tumors (Figure 8d). Additional assessment of other OXPHOS complex subunits indicated that NDUFA1 (Complex I) was also upregulated in resistant tumors, whereas no significant differences were detected in SDHB (Complex II) or ATP5O (Complex V) (Figure S11a,b), highlighting the specific involvement of Complex I in platinum resistance.

We next evaluated the clinical relevance of CD19^+^ B cell infiltration and NDUFA8 expression using pre‐treatment biopsies from an expanded cohort of patients enrolled in the SAT1 clinical trial (Zhongshan Hospital, B2022‐087(5)). Chemotherapy responsiveness was quantified using the modeled CA‐125 elimination rate constant (KELIM), a validated kinetic parameter reflecting intrinsic sensitivity to platinum‐based therapy. CD19^+^ B cell infiltration showed a significant positive correlation with KELIM scores, indicating enhanced chemotherapy responsiveness in tumors enriched with B cells (R^2^ = 0.1233, p = 0.0055, Figure 8e,f). Consistently, patients classified as KELIM‐high (≥1) exhibited significantly greater CD19^+^ B cell abundance compared with KELIM‐low (<1) tumors (Figure S11c). Receiver operating characteristic (ROC) analysis demonstrated a moderate discriminatory capacity, with an area under the curve (AUC) of 0.7, supporting its ability to distinguish KELIM‐defined sensitive and resistant cases (Figure 8i). Conversely, NDUFA8 expression displayed a significant negative correlation with KELIM, consistent with an association between elevated Complex I‐related mitochondrial bioenergetic activity and reduced platinum sensitivity(R^2^ = 0.16, p = 0.001, Figure 8g,h). Stratified analysis further confirmed higher NDUFA8 expression in KELIM‐low tumors (Figure S11d). ROC analysis similarly yielded an AUC of 0.73, indicating a comparable moderate ability to classify chemotherapy response status (Figure 8i). Collectively, these findings demonstrate that CD19^+^ B cell‐associated immune contexture and NDUFA8‐mediated mitochondrial bioenergetic status are significantly correlated with platinum responsiveness in advanced high‐grade serous ovarian cancer and exhibit moderate discriminatory performance, supporting their potential utility as response‐associated biomarkers.

## 3 Discussion

Neoadjuvant chemotherapy (NACT) provides a clinically informative setting to interrogate intrinsic tumor chemosensitivity through integration of multi‐omics profiling with treatment outcomes. In this study, we demonstrate that NACT reshapes the tumor microenvironment toward an immune‐activated state. Integrative analyses further identify two opposing biological programs associated with therapeutic response: an immune‐enriched state characterized by increased CD19^+^ B‐cell infiltration linked to favorable sensitivity, and a metabolically rewired state marked by elevated mitochondrial Complex I activity and NDUFA8 expression associated with resistance. These findings suggest that pretreatment B‐cell abundance may aid patient stratification, while Complex I‐dependent bioenergetic stability represents a targetable metabolic vulnerability. Collectively, our data support a dual‐axis model in which immune activation and metabolic dependency jointly determine chemotherapy responsiveness in high‐grade serous ovarian cancer.

Immune activation represents a prominent feature of NACT‐induced tumor microenvironment remodeling. Previous multi‐omics studies in HGSOC have shown that chemotherapy enhances cytotoxic T‐cell expansion, increases NK‐cell infiltration, modulates myeloid populations, and reshapes antigen presentation programs, while also inducing context‐dependent T‐cell exhaustion [12, 14, 15, 16]. Consistent with these observations, we found augmented immune infiltration in post‐NACT tumors, including increased plasma B cells, memory CD4^+^ T cells, activated dendritic cells, CD8^+^ T cells, and NK cells. Notably, distinct chemotherapeutic regimens may differentially influence immune remodeling. In our cohort, nab‐paclitaxel‐based NACT was associated with enhanced plasma B‐cell infiltration and complement activation compared with conventional paclitaxel. Collectively, these findings suggest that chemotherapy‐induced immune reprogramming may provide a biological rationale for combination strategies with immunotherapy.

The Chemotherapy Response Score (CRS) and KELIM are established indicators of platinum sensitivity and survival in HGSOC [17, 18]. By integrating these complementary metrics across independent cohorts, we identified CD19^+^ B‐cell infiltration as a consistent immune correlate of neoadjuvant chemotherapy responsiveness. This multi‐omics framework highlights the relevance of B‐cell abundance as a stratification feature associated with treatment sensitivity. B lymphocytes are key components of the tumor microenvironment and contribute to antitumor immunity through antigen presentation and coordination with T cells, NK cells, and myeloid populations [19]. High levels of tumor‐infiltrating B cells have been linked to favorable outcomes across multiple malignancies, including HGSOC and breast cancer [20, 21, 22, 23]. Consistent with this body of evidence, the infiltration of CD19^+^ B‐cell in our cohort was correlated with improved survival and therapeutic response. While CD20 is commonly used to identify B cells within tertiary lymphoid structures, emerging data support CD19 as a sensitive and clinically relevant marker of treatment‐associated immune activation [24, 25], reinforcing its potential utility in response stratification models.

In parallel, we identified hyperactivated oxidative phosphorylation (OXPHOS), particularly Complex I‐associated signaling, as a metabolic feature of platinum resistance. Elevated mitochondrial respiration has been widely linked to metastasis, stem‐like properties, and therapy resistance across malignancies, and pharmacologic inhibition of mitochondrial energetics can sensitize tumors to chemotherapy [26, 27, 28, 29]. Consistently, increased expression of Complex I components, including NDUFA8/NDUFB8, has been associated with resistant phenotypes [30, 31]. Although prior work has reported improved chemotherapy response in certain high‐OXPHOS HGSOC [32], such discrepancies may reflect differences in classification strategies. Whereas earlier studies relied primarily on metabolic gene expression, our analysis integrated CRS‐based pathological response with multi‐omics profiling, capturing broader biological determinants of treatment sensitivity. Mechanistically, Complex I inhibition disrupts mitochondrial bioenergetic stability, inducing mitophagy through membrane depolarization and PINK1 activation. Although autophagy is often considered cytoprotective, its functional impact is highly context‐dependent. In certain settings, activation of autophagic flux can enhance chemotherapy sensitivity by dismantling pro‐survival signaling networks, whereas in metabolically flexible tumors it may sustain ATP production and redox balance, thereby promoting resistance [33]. Our data suggest that in platinum‐resistant ovarian cancer cells with strong Complex I dependency, mitochondrial stress instead exposes an energetic vulnerability, lowering the threshold for DNA damage tolerance. Thus, mitochondrial quality control emerges as a context‐dependent regulator of platinum sensitivity. While this model provides a coherent mechanistic explanation linking mitochondrial dysfunction to therapeutic response, additional molecular details governing this process warrant further investigation in future studies.

Therapeutically, our data support metabolic intervention as a potential strategy for high‐OXPHOS tumors. However, systemic inhibition of electron transport chain components has been limited by toxicity in clinical development (NCT02882321, NCT03291938) [34]. Rather than broad ETC blockade, selective targeting of resistance‐associated nodes‐such as NDUFA8, whose suppression sensitized resistant cells in our models, may represent a more precise and tolerable approach. Moreover, emerging evidence suggests mitochondrial metabolism intersects with immune regulation [35, 36], raising the possibility that immune‐metabolic co‐targeting strategies could enhance therapeutic efficacy.

This study is limited by sample size, particularly for paired multi‐omics and organoid analyses. Although clinical validation was confined to HGSOC, some in vitro experiments utilized ovarian cancer cell lines that do not fully recapitulate canonical TP53‐driven features. Nevertheless, the central conclusions linking CD19^+^ B‐cell infiltration to chemotherapy sensitivity and Complex I‐associated metabolic reprogramming to resistance were derived from and independently validated in clinical specimens. Additionally, our study reveals that combination therapy triggers excessive mitophagy, which acts as a key upstream driver of DNA damage‐mediated resensitization. This causal link is confirmed by the fact that Chloroquine (CQ) significantly rescues the DNA damage and apoptotic phenotype. While the precise molecular intermediates linking mitophagy to DNA repair impairment remain to be fully characterized, representing a limitation of the current study, it is highly plausible that this synergy stems from a mitophagy‐induced bioenergetic crisis or metabolic‐redox imbalance that disables the energy‐dependent DNA repair machinery. Although a full‐scale dissection of these systemic metabolic interactions is beyond our present scope, the identification of this functional axis provides a compelling mechanistic framework for overcoming platinum resistance in ovarian cancer.

In summary, our findings delineate complementary immune and metabolic determinants of neoadjuvant chemotherapy response in ovarian cancer. CD19^+^ B‐cell infiltration and NDUFA8‐associated mitochondrial bioenergetics together define an immune‐metabolic axis that shapes platinum sensitivity, providing a conceptual framework for stratification and therapeutic optimization.

## 4 Methods

### 4.1 Cohorts and Specimens

The study was established based on the clinical trial ChiCTR1900026893. The trial design was detailed in the previous study [11]. To compare the influence of nab‐paclitaxel and paclitaxel on the microenvironment, 23 patients who received NACT of paclitaxel and carboplatin were enrolled in the study. Inclusion criteria were consistent with the above trial. Pre‐treatment and post‐treatment tissues were collected by laparoscopy before the NACT treatment and debulking surgery for transcriptomic, metabolic, proteomics, and multiplex immunohistochemistry (mIHC). The samples were collected and stored in RNA later for transcriptomic at liquid nitrogen for metabolic, proteomics, and mIHC. Due to limited samples obtained from laparoscopes, 19 samples with the same population but conducting primary debulking surgery were added as pre‐treatment tissues. All participants provided written informed consent. The genomic status was obtained by the department of molecular pathology that every ovarian cancer patient routinely performs genetic testing to get the mutation status of BRCA1/2 or other DNA repair‐related genes. For paired samples, the detected tissues were post‐NCAT.

An independent validation cohort was derived from the SAT‐1 clinical study at Zhongshan Hospital, Fudan University. The SAT‐1 trial is a multi‐center, randomized, phase II study evaluating the efficacy of different surgical timings in combination with targeted therapy for advanced ovarian cancer in China. It enrolled patients with FIGO stage IIIC‐IV disease who were assessed as eligible for achieving satisfactory tumor reduction. From this trial, pretreatment biopsy samples were collected from 65 patients undergoing NACT. These samples were analyzed to assess CD19^+^ B cell infiltration levels and NDUFA8 protein expression in relation to chemotherapy response.

### 4.2 RNA Extraction, Sequencing and Transcriptomic Analysis

Total RNA extraction and RNA‐seq libraries were performed and prepared using the VAHTS Universal RNA‐Seq Library Prep Kit for Illumina (Vazyme), followed by sequencing on an Illumina NovaSeq 6000 platform as previously described [37]. Raw reads were subjected to quality control using FastQC. Clean reads were aligned to the human reference genome (GRCh38) using the STAR aligner. Gene‐level counts were generated using featureCounts based on GENCODE v29 annotation. To account for potential technical variability across sequencing lanes, batch effects were adjusted using the removeBatchEffect function in limma. Differential expression and functional enrichment analyses were conducted with thresholds of |log2FC| ≥1 and FDR <0.05.

### 4.3 Metabolic Sequencing and Analysis

Frozen tissues from 12 paired pre‐ and post‐NACT samples were homogenized in 80% methanol (1:10, w/v), followed by ultrasonication for 30 min and centrifugation at 11 000 rpm for 30 min at 4°C. The supernatant was collected, lyophilized, and reconstituted in an appropriate solvent for LC‐MS/MS analysis. Metabolomic profiling was performed on a Vanquish UHPLC system coupled to an Orbitrap Eclipse Tribrid mass spectrometer (Thermo Fisher Scientific). Chromatographic separation was achieved using a Waters ACQUITY BEH C18 column (100 × 2.1 mm, 1.7 µm) with a 20‐min linear gradient of 0.1% formic acid in water (A) and acetonitrile (B) at a flow rate of 0.4 mL/min. The electrospray ionization (ESI) voltage was set to 3.5 kV, and full MS scans were acquired over an m/z range of 150–2000. To ensure analytical stability and minimize technical variation, pooled quality control (QC) samples were prepared by mixing equal aliquots from all samples and injected periodically throughout the analytical run. Sample injection order was randomized to reduce potential systematic bias. Raw data were processed using Compound Discoverer 3.3 for peak detection, alignment, and metabolite annotation. Peak intensities were normalized using the QC‐based robust LOESS signal correction (QC‐RLSC) method implemented in the StatTarget R package to correct for signal drift and batch effects. Data were log2‐transformed and Pareto‐scaled prior to multivariate analysis. Principal component analysis (PCA) and orthogonal partial least squares discriminant analysis (OPLS‐DA) were performed using SIMCA software to assess global metabolic differences. Differentially expressed metabolites (DEMs) were identified based on variable importance in projection (VIP) > 1, false discovery rate (FDR) < 0.05 (Student's *t*‐test), and |log2 fold change| > 1.5. Pathway enrichment analysis was conducted using MetaboAnalyst 6.0, with thresholds of pathway impact > 0.1 and adjusted p‐value < 0.05.

### 4.4 Proteomics Sequencing and Analysis

For proteomic analysis, samples were homogenized in RIPA buffer and centrifuged at 12,000 rpm, 4°C for 30 min. The supernatant was collected, and protein concentration was quantified. Proteins were then reduced (DTT, 37°C, 2 h), alkylated (IAM, 30 min, dark), trypsin‐digested (37°C, overnight), and vacuum‐dried for LC‐MS/MS. Liquid chromatography was performed using an Agilent 1100 Series system coupled to a Triple TOF 6600 mass spectrometer. For spectral library generation, data‐dependent acquisition (IDA) mode was employed, including TOF‐MS survey scans followed by MS/MS fragmentation of precursor ions. For quantitative analysis, data‐independent acquisition (SWATH‐MS) was performed, and peak extraction and quantification were conducted using MultiQuant software. All samples were analyzed in a single continuous acquisition run to minimize technical variability. Raw intensity values were log2‐transformed. Principal component analysis (PCA) was performed to evaluate overall data. Differentially expressed proteins were identified using paired Student's *t*‐test with thresholds of |log2 fold change| > 1.5 and p‐value < 0.05, followed by Benjamini–Hochberg false discovery rate (FDR) correction.

### 4.5 Human Ovarian Cancer Cell Lines and Transfection

Human epithelial ovarian cancer cell lines OVCA‐433, HEY, OVCA‐429, SKOV3, OVCA‐R8 and OVCA‐R3 were purchased from American Type Culture Collection (ATCC). Cells were cultured in DMEM (Viva Cell, Cat #C3113), supplemented with 10% fetal bovine serum (Viva Cell, Cat #C3420), penicillin (100 U/mL) and streptomycin (100 µg/mL; Viva Cell, Cat #C04001) in a humidified atmosphere of 5% (v/v) CO2 in air at 37°C. All human cell lines were verified to be free of mycoplasma contamination.

All small interfering RNA (siRNA) and the corresponding negative control (NC) were chemically synthesized by GeneCreate (Wuhan, China). The transfections were conducted using Lipofectamine 3000 (Yeasen Biotechnology, Shanghai, China) as a transfecting agent as described previously [38]. The sequences of siRNA were listed in Table S2.

### 4.6 The Generation and Drug Screening of Patient‐Derived Organoids

6 patient‐derived organoids (PDOs) were established from primary debulking surgical tumor specimens obtained with informed consent. All patients were staged at IIIC‐IV. Tissues were cleaned with PBS and dissociated using a mixture of 0.5 U dispase and collagenase I at 37 °C for 30–60 min. Digestion was terminated by adding an equal volume of DMEM supplemented with 10% serum. The resulting suspension was filtered through a 100 µm strainer, centrifuged at 1500 rpm for 5 min, and resuspended in Matrigel. After solidification for 20 min, culture medium was added and refreshed every 2–3 days. Organoids were passaged every 1–4 weeks at a 1:2‐3 ratio. For histological analysis, organoids were fixed in 4% paraformaldehyde, embedded in paraffin, sectioned, and stained with H&E or immunohistochemical markers. Two pathologists independently evaluated the cellular composition of all organoid models.

For drug assays, organoids were dissociated and seeded in Matrigel at 2000–5000 cells per well in 7 µL drops. After 30 min of incubation, 100 µL of culture medium was added to each well. Drug treatment was initiated 72 h post‐seeding or when significant organoid growth was observed. Prepared compounds were equilibrated to room temperature, vortexed, and added at designated concentrations following the removal of 50 µL spent medium. After 144 h of drug exposure, viability was assessed using CellTiter‐Glo reagent. Luminescence signals were measured with a microplate reader, and dose‐response curves were analyzed using GraphPad Prism.

### 4.7 Seahorse Technology

The oxygen consumption rate (OCR) and the extracellular acidification rate (ECAR) were evaluated using the Seahorse XF Cell Mito stress test kit (Agilent, #103708‐100) and Seahorse XF Glycolysis Stress Test Kit (Agilent, #103710‐100) in the Seahorse XFe 96 Extracellular Flux Analyzer (Seahorse Bioscience, USA) according to the manufacturer's instructions. Briefly, individual groups of cells (6 × 10^3^ cells/well) were cultured into a Seahorse XF 96‐well microplate, and their baseline level were determined. Then, the cells were treated sequentially with glucose, oligomycin, and 2‐DG at the indicated doses and time points for measurement of ECAR. Similarly, the cells were treated sequentially with oligomycin, oxidative phosphorylation FCCP, and rotenone/antimycin A. Data were analyzed by Seahorse XF‐96 Wave software and expressed as pmols/min for OCR and mpH/min for ECAR, respectively.

### 4.8 Mitochondrial Staining Using MitoTracker Probe

For mitochondrial imaging, cells plated on glass coverslips were incubated with 100 nM MitoTracker Red CMXRos (Beyotime Biotechnology, Cat# C1035) in complete medium at 37°C for 30 min. Cells were then fixed with 4% paraformaldehyde (PFA) for 15 min at room temperature, permeabilized with 0.5% Triton X‐100 for 10 min, and blocked with 5% bovine serum albumin (BSA) for 1 h. Nuclei were stained with DAPI (1 µg/mL) for 5 min. Coverslips were mounted using ProLong Diamond Antifade Mountant (Thermo Fisher, Cat# P36965). Images were acquired on a Zeiss LSM 900 confocal microscope with a 63× oil‐immersion objective (NA 1.4) using 405 nm (DAPI) and 561 nm (MitoTracker Red) laser lines. Z‐stacks were collected at 0.3 µm intervals. Mitochondrial fluorescence intensity was quantified in >50 cells per group using ImageJ v1.53 with background subtraction (rolling ball radius: 50 pixels), normalized to nuclear DAPI area.

### 4.9 Mitochondrial Membrane Potential(MMP)Detection by Flow Cytometry

Mitochondrial membrane potential was assessed using the Mitochondrial Membrane Potential and Apoptosis Detection Kit with Mito‐Tracker Red CMXRos and Annexin V‐FITC (Beyotime Biotechnology, Cat# C1071M). Briefly, culture medium was aspirated into a centrifuge tube. Adherent cells were washed once with PBS, detached using trypsin‐EDTA solution, and resuspended in the collected medium. After centrifugation at 1000 rpm for 5 min, cells were counted. Approximately 1 × 10^5^ cells were gently resuspended in 188 µL of Annexin V‐FITC binding buffer. Subsequently, 2 µL of Mito‐Tracker Red CMXRos staining solution, 5 µL of Annexin V‐FITC, and 5 µL of Hoechst 33342 staining solution were added. Cells were incubated at room temperature in the dark for 30 min and analyzed by flow cytometry. Fluorescence detection parameters were Mito‐Tracker Red CMXRos (Ex/Em: 579/599 nm, red), Annexin V‐FITC (Ex/Em: 492/520 nm, green), and Hoechst 33342 (Ex/Em: 350/461 nm, blue).

### 4.10 Electron Microscopy

Following treatment, cells cultured in 10 cm dishes were detached using trypsin, and the resulting cell pellet was resuspended in TEM fixative (Servicebio, G1102) for 30 min at room temperature protected from light. The sample was then post‐fixed in 1% osmium tetroxide (Ted Pella Inc, 18456) prepared in 0.1 M phosphate buffer (PB, pH 7.4) for 2 h at room temperature protected from light. After three 15 min washes with 0.1 M PB (pH 7.4), the sample was dehydrated at room temperature through a graded ethanol series (30%, 50%, 70%, 80%, 95%, and twice in 100%; Sinaopharm Group Chemical Reagent Co. LTD, 100092183), each step for 20 min, followed by two 15 min washes in 100% acetone (Sinaopharm Group Chemical Reagent Co. LTD, 10000418). Infiltration and embedding were performed sequentially: a 1:1 mixture of acetone to EMBed 812 resin (SPI, 90529‐77‐4) for 2–4 h at 37°C, followed by a 1:2 mixture of acetone to resin overnight at 37°C, and finally pure EMBed 812 resin for 5–8 h at 37°C. The sample was then placed in pure EMBed 812 resin (with catalyst) within an embedding mold and incubated overnight at 37°C. Polymerization was carried out by transferring the mold to a 60°C oven for 48 h. The resulting resin block was sectioned to 60–80 nm thickness using an ultramicrotome (Leica UC7) equipped with a diamond knife (Diatome, Ultra 45°), and sections were collected onto 150‐mesh copper grids with formvar film. Grids were stained with 2% uranyl acetate in saturated ethanol for 8 min, protected from light, rinsed three times with 70% ethanol and three times with ultrapure water, stained with 2.6% lead citrate (prepared with CO_2_ protection) for 8 min, and rinsed three times with ultrapure water. Excess liquid was gently removed with filter paper, and the grids were air‐dried overnight at room temperature. Samples were examined, and images were acquired using a transmission electron microscope (HITACHI HT7800/HT7700).

### 4.11 Reduced Glutathione (GSH) Assay

Intracellular GSH levels were measured using the GSH and GSSG Assay Kit (Beyotime Biotechnology, Cat# S0053). Working solutions were prepared as follows: (a) Protein Removal Reagent M: 0.2 g Reagent M dissolved in 4 mL total glutathione assay buffer; (b) Total Glutathione Detection Working Solution: Prepared per sample using 6.6 µL of 5× diluted glutathione reductase, 6.6 µL of DTNB stock solution, and 150 µL of total glutathione assay buffer; (c) *0.5 mg/mL NADPH*: 10 µL NADPH stock solution diluted in 790 µL total glutathione assay buffer; (d) Diluted GSH Scavenging Auxiliary Solution: 53 µL GSH scavenging auxiliary solution mixed with 47 µL ddH_2_O; (e) GSH Scavenging Working Solution: 10.8 µL GSH scavenging reagent mixed with 89.2 µL absolute ethanol. A 10 mM GSSG stock solution was diluted with Reagent M to prepare a 15 µM standard solution, followed by serial dilution to generate the standard curve.

Cells were seeded, treated with radiation or drugs for 48 h, and pelleted. Cell pellets were lysed with 150 µL of Protein Removal Reagent M, subjected to two freeze‐thaw cycles (liquid nitrogen followed by a 37°C water bath), incubated on ice for 5 min, and centrifuged at 10 000 × g for 10 min at 4°C. The supernatant was collected for analysis. For measurement, 10 µL of sample or standard and 150 µL of detection working solution were mixed in a 96‐well plate. After 5 min incubation at room temperature, 50 µL of NADPH solution was added. Absorbance at 412 nm (A412) was measured every 5 min for 25 min (5 readings total) using a microplate reader. Data were analyzed using GraphPad Prism with triplicate wells per condition.

### 4.12 NAD+/NADH Assay

The NAD^+^/NADH Assay Kit with WST‐8 (Beyotime Biotechnology, Cat# S0175) was used. Harvested cells were counted, and 1 × 10^6^ cells were lysed in 200 µL of NAD^+^/NADH extraction buffer, followed by centrifugation at 12 000 × g for 10 min at 4°C. The supernatant was collected as the test sample. A 10 mM NADH standard stock solution was prepared by dissolving 5 mg NADH in 655 µL NADH preparation buffer and serially diluted with NAD^+^/NADH extraction buffer to concentrations of 0, 0.25, 0.5, 1, 2, 4, 6, 8, 10, and 30 µM. Alcohol dehydrogenase (ADH) working solution was prepared by diluting ADH 45‐fold in reaction buffer. For measurement, 20 µL of sample or standard was added to a 96‐well plate, incubated at 37°C in the dark for 10 min, mixed with 10 µL of chromogenic solution, and further incubated at 37°C in the dark for 20 min. Absorbance at 450 nm (A450) was measured to determine total NAD^+^ and NADH levels. To measure NADH specifically, samples were heated at 60°C for 30 min in a PCR instrument to decompose NAD^+^, followed by the same assay procedure. NAD^+^ levels and the NAD^+^/NADH ratio were calculated. Data were analyzed using GraphPad Prism.

### 4.13 IC_50_ Determination Assay

IC_50_ assay was used to evaluate the sensitivity of ovarian cancer cells to carboplatin and the synergistic effect of an ETC inhibitor on carboplatin. Cells were seeded in 96‐well flat‐bottom plates at a density of 2500 cells/well in 100 µL complete medium. After 24 h of adhesion, cells were exposed to serial dilutions of drugs with 8 concentrations according to 3 fold dilutions in medium. Drugs used in the study were listed as follow: Carboplatin (MCE, HY‐17393) with the beginning concentration of 6.25 uM, three Complex I inhibitors including IACS‐010759 (MCE, HY‐112037) with the beginning concentration of 2.5 uM, Rotenone (MCE, HY‐B1756) with the beginning concentration of 0.123 uM, VLX600 (MCE, HY‐12406) with the beginning concentration of 3.9 nM, Complex III inhibitor Antimycin V (Maokang Bio, MS0070) with the beginning concentration of 3.75 uM and Complex V inhibitor Gboxin (MCE, HY‐111651) with the beginning concentration of 3.125 uM. Following 72 h incubation at 37°C, 10 µL CCK‐8 reagent (Lablead, Cat# CK001) was added per well. Plates were incubated for 2 h at 37°C, and absorbance was measured at 450 nm using a microplate reader (BioTek Synergy H1). Cell viability was calculated as: Viability (%) = [(A_treated—A_blank) / (A_control—A_blank)] × 100. IC_50_ values were calculated by nonlinear regression using a four‐parameter logistic model [log(inhibitor) vs. normalized response – variable slope] in GraphPad Prism. To meet normality assumptions for statistical comparison, IC_50_ values were log10‐transformed before analysis. Log10(IC_50_) between groups was compared using the appropriate test as indicated in figure legends.

### 4.14 Combination Index (CI) Calculation

Drug interactions were quantified by the Chou‐Talalay method: The combination index (CI) was calculated as CI = (D_1_/D_x__1_) + (D_2_/D_x__2_), where D_1_ and D_2_ are the doses of drug 1 and drug 2 in combination that achieve a specified effect level, D_x__1_ nd D_x__2_ are the doses of drug 1 and drug 2 alone that achieve the same effect level(e.g., 50% inhibition). Interpretation: CI < 1 (synergy), CI = 1 (additivity), CI > 1 (antagonism).

### 4.15 qRT‐PCR of ETC Genes from Cell Lines

Quantitative real—time polymerase chain reaction (qRT‐PCR) was used for the electron transport chain (ETC) gene expression analysis. Total RNA was extracted using the Super‐Fast Pure Cell RNA Isolation Kit (Vazyme, RC102). RNA concentration and purity were determined using a NanoDrop spectrophotometer (NanoDrop Technologies). Subsequently, 1 µg of total RNA was reverse transcribed into cDNA using the FastKing RT Kit (Vazyme, AG11728). qRT‐PCR was performed with Power SYBR Green Master Mix (Vazyme, AG11718) on a Chromo4 Real‐Time PCR Detection System (Bio‐Rad). Gene‐specific primers were used at a final concentration of 300 nM (sequences provided in Table S2). All reactions were performed in triplicate. Expression levels of target genes were normalized to the endogenous reference gene CYCLOPHILIN‐B and calculated as fold change relative to controls using the 2^(‐ΔΔCt) method.

### 4.16 Multiplexed Immunohistochemistry Assay

A multiplex immunofluorescence assay was used to determine the infiltration of various kinds of immune cells, which was performed using a previous protocol [39].

### 4.17 Animal Model and B Cell Flow Cytometry Analysis

All animal experiments were approved by the Institutional Animal Care and Use Committee of Fudan University (FUSCC‐IACUC‐2025233). A total of 1×10^7^ human ovarian cancer cells (A2780, SKOV‐3, OVCA‐R8, and HEY) suspended in 100 µL of PBS were subcutaneously inoculated into each female nude mouse (4 weeks old, Shanghai), with five replicates per cell line. Treatment was initiated when tumor volumes reached approximately 1500 mm^3^. The tumor volumes were calculated using the formula (longest diameter) × (shortest diameter)^2^ × 0.5 and presented as mean ± standard error of the mean (SEM). For platinum‐resistant cell lines (OVCA‐R8 and HEY), tumor‐bearing mice were divided into four treatment groups: an untreated control group; a carboplatin group receiving intraperitoneal injection of carboplatin (25 mg/kg, three times per week); an IACS‐010759 group administered IACS‐010759 (5 mg/kg via oral gavage, three times weekly); and a combination group receiving both agents concurrently. After 21 days of treatment, mice were euthanized, and tumors were harvested. Tumor tissues were paraffin‐embedded, sectioned, and subjected to immunohistochemical staining using anti‐Ki67 and anti‐γH2AX antibodies to evaluate cell proliferation and DNA damage response, respectively.

For platinum‐sensitive cell lines (A2780 and SKOV3), fresh tumor tissues were collected and processed for flow cytometric analysis of B‐cell infiltration. Tissues were minced into 2–3 mm^3^ fragments and digested with collagenase IV for 2 h at 37 °C. The resulting single‐cell suspensions were filtered through 100 µm strainers, followed by red blood cell lysis using ACK buffer. Cells were stained with Zombie (Dakewe Biotech, Shenzhen, China,1:1000) viability dye and fluorochrome‐conjugated antibodies against CD45, CD3, and CD19. CD19^+^ leukocytes were gated to identify CD45^+^CD19^+^ B lymphocytes. All antibodies were purchased from Dakewe Biotech and diluted as follows: CD45(1:80), CD3(1:50), CD19(1:160).

### 4.18 Immunohistochemical Staining (IHC)

Immunohistochemistry (IHC) was used to evaluate the expression levels of specific molecules, such as NDUFA8 and CD19, in tumor tissues, following previously described protocols [39]. For external validation, 65 pre‐NACT tumor biopsy samples were obtained from Zhongshan Hospital, Fudan University. CD19 staining was successfully performed on 61 specimens due to insufficient tumor‐infiltrating lymphocytes in the remaining 4 needle biopsy samples, where tumor cells were present but lacked a detectable lymphocytic infiltrate, while NDUFA8 staining was successfully completed for all 65 cases. Referring to the method for assessing tumor‐infiltrating lymphocytes (TILs) [40], we analyzed the infiltration level of CD19^+^ B cells and performed correlation analyses with the KELIM index. In brief, tumor stromal regions were first identified through hematoxylin and eosin (H&E) staining, and the proportion of CD19^+^ B cells was subsequently analyzed within these stromal areas. Quantification of CD19^+^ B cells was performed using fully automated digital image analysis. Whole‐slide images were acquired using a Pannoramic 250 FLASH scanner (3DHISTECH) and analyzed with custom algorithms in Image Pro Plus. Tumor regions were first manually annotated to exclude necrotic or non‐tumor areas, after which the algorithm quantified the CD19^+^ fluorescent area as a percentage of total viable tumor area. Signal thresholding parameters were predefined and applied uniformly across all samples to ensure consistency. As analysis was fully automated, inter‐observer variability was minimized; tumor region annotation was performed by two independent investigators blinded to clinical outcomes. All primary antibodies were purchased from Abcam and diluted as follows: NDUFA8 (1:400), NDUFA1(1:400), SHDB (1:400), ATP5O (1:400), Ki‐67(1:200), and γH2AX (1:1000). The immunohistochemical staining results were evaluated using the H‐score method to quantify protein expression levels. The H‐score was calculated by assigning intensity scores as follows: 0 (negative), 1 (weak), 2 (moderate), and 3 (strong), and multiplying the percentage of cells at each intensity level by the corresponding score, followed by summing these values, with the formula H‐score = (3 × percentage of strong positive cells) + (2 × percentage of moderate positive cells) + (1 × percentage of weak positive cells), yielding a range of 0 to 300. Two independent pathologists, blinded to the clinical data, performed the scoring, and discrepancies were resolved through consensus review.

### 4.19 Mitochondria and Lysosome Co‐staining Analysis

Cells seeded in confocal dishes were treated as indicated. For co‐staining of mitochondria and autophagic vacuoles, cells were first incubated with 100 nM MitoTracker Red CMXRos (Beyotime Biotechnology, Cat# C1035) in complete medium at 37°C for 30 min to label mitochondria. Subsequently, without fixation, cells were stained with 50 µM MDC (Lyso‐Tracker Green, Beyotime Biotechnology, Cat# C1047S) in complete medium at 37°C for 30 min in the dark according to the manufacturer's instructions for live‐cell autophagic vacuole labeling. After staining, live cells were immediately imaged using a Zeiss LSM 900 confocal microscope with a 10× objective. Lyso‐Tracker Green fluorescence was excited at 405 nm, and emission was collected between 510 and 540 nm, while MitoTracker Red fluorescence was excited at 561 nm. Z‐stacks were collected at 0.3 µm intervals. For quantitative analysis, the mean fluorescence intensities of MitoTracker and Lyso‐Tracker were measured in >30 cells per group using ImageJ v1.53 with background subtraction (rolling ball radius: 50 pixels). Statistical analysis was performed using GraphPad Prism, with significance determined by unpaired *t*‐test or one‐way ANOVA as appropriate.

### 4.20 Comet Assay

The Alkaline Comet Assay was performed to assess cellular DNA damage(ab238544, Abcam). Briefly, treated cells (1 × 10^5^ cells/mL) were mixed with 0.5% low‐melting‐point agarose (LMPA) and spread onto slides pre‐coated with 1% normal‐melting‐point agarose (NMPA). Following lysis in a chilled buffer (2.5 M NaCl, 100 mM EDTA, 10 mM Tris, 1% Triton X‐100, pH 10.0) at 4°C for 2 h, the slides were placed in an alkaline electrophoresis solution (300 mM NaOH, 1 mM EDTA, pH > 13) for 20 min to allow for DNA unwinding. Electrophoresis was conducted at 25 V and 300 mA for 20 min. After neutralization with 0.4 M Tris‐HCl (pH 7.5), DNA was stained with a Vista Green DNA Dye (10 000×, diluted as required) and visualized under a fluorescence microscope. At least 50 randomly selected cells per group were analyzed using Comet Assay software, with the Tail Moment and % Tail DNA used as indicators of DNA fragmentation.″

### 4.21 Western Blot Analysis

Cells were harvested and lysed in RIPA buffer (Beyotime, Cat# P0013B) supplemented with protease and phosphatase inhibitor cocktails. Protein concentrations were determined using a BCA protein assay kit (Beyotime, Cat# P0012). Equal amounts of protein (20–30 µg) were separated by SDS‐PAGE using a rapid electrophoresis buffer system (Servicebio, Cat# G2081‐1L) and then transferred onto PVDF membranes using the Omni‐Flash ice‐bath‐free rapid transfer buffer (10×, epizyme, Cat# PS201S). Membranes were blocked with 5% non‐fat milk in Tris‐buffered saline containing 0.1% Tween‐20 (TBST) for 1 h at room temperature, followed by overnight incubation at 4°C with primary antibodies against the target proteins (see detailed antibody list below). After washing with TBST, membranes were incubated with horseradish peroxidase (HRP)‐conjugated secondary antibodies for 1 h at room temperature. Protein bands were visualized using an enhanced chemiluminescence (ECL) detection system.

Antibodies Used for Western Blotting:

PINK1 (Affinity, Cat# DF7742, dilution 1:3000)
p‐ATR (Ser428) (Affinity, Cat# DF7512, dilution 1:1000)
BAX (Affinity, Cat# AF0120, dilution 1:4000)
Bcl‐2 (Affinity, Cat# AF6139, dilution 1:3000)
Phospho‐Histone H2A.X (Ser139) (Affinity, Cat# AF3187, dilution 1:5000)
RAD51 (Affinity, Cat# DF8066, dilution 1:1000)
LC3A/B (Affinity, Cat# AF5402, dilution 1:3000)
Tubulin alpha (Affinity, Cat# AF7010, dilution 1:10000)

### 4.22 Statistical Analysis

All statistical analyses were performed using GraphPad Prism version 9.0 (GraphPad Software, San Diego, CA, USA). Normality was assessed using the Shapiro‐Wilk test. Homogeneity of variances was assessed using Levene's test. Quantitative data are expressed as mean ± standard deviation (SD) or mean ± standard error of the mean (SEM), as specified in the corresponding figure legends, with the exact sample sizes (n) detailed in the legends. Comparisons between two groups were conducted using two‐tailed unpaired Student's *t*‐tests, as appropriate for the experimental design specified in each figure legend. For multiple group comparisons, one‐way analysis of variance (ANOVA) followed by Tukey's multiple comparisons test was used (alpha = 0.05). For two‐way ANOVA, Tukey's multiple comparisons test was applied to compare all pairs of cell type and treatment combinations. Survival outcomes were evaluated using Kaplan‐Meier curves, and differences between groups were assessed by the log‐rank test. Receiver operating characteristic (ROC) curve analysis was performed to evaluate the predictive performance of CD19 and NDUFA8, with the area under the curve (AUC) calculated to assess their discriminatory capacity. A p‐value of less than 0.05 was considered statistically significant. Significance levels are indicated in the figures as follows: ^*^ *p* < 0.05, ^**^ *p* < 0.01, ^***^ *p* < 0.005, ^****^ *p* < 0.001; ns, not significant. All error bars represent mean ± SD or mean ± SEM, as indicated in each figure legend.

## Author Contributions

H.J.Y contributed to conceptualization and project coordination. W.J contributed to study design and manuscript writing with the help of all authors. Y.W performed data analysis and contributed to the design and execution of a subset of cellular experiments. X.H.L carried out cellular and animal experimental procedures. Q.L.Y provided genetic testing analysis and interpretation. L.B.X, as the principal clinical investigator of the SAT1 study at Zhongshan Hospital, contributed pre‐neoadjuvant therapy patient samples. X.C, X.Y.T, and S.X.T contributed tissue sectioning, CD19 immunohistochemical staining, and scoring. Y.F.C performed CRS evaluation and CD19 evaluation. L.M.S was responsible for metabolomic and proteomic profiling of samples. Y.X assisted with the analysis of the sequencing data.

## Ethics Statement

The study was approved by the Ethics Committee of Fudan University Shanghai Cancer Center (FUSCC 050432‐4‐1212B) and conducted in accordance with the approved guidelines.

## Conflicts of Interest

The authors declare no conflict of interest.

## Supporting information




**Supporting file 1**: advs76612‐sup‐0001‐SuppMat.docx

**Supporting file 2**: advs76612‐sup‐0002‐DataFile.zip

## Data Availability

The data that support the findings of this study are available from the corresponding author upon reasonable request.
