# Supplementary material for: Integrative Multi‐Omics Analysis Reveals a Mitochondrial–Immune Axis Associated With Neoadjuvant Chemotherapy Response in High‐Grade Serous Ovarian Cancer
Source: Adv Sci (Weinh). 2026 Jul 20;13(56):e23029. doi: 10.1002/advs.202523029 (PMC13383156; doi:10.1002/advs.202523029)
Supplement: Supplementary file 1 [file ADVS-13-e23029-s001.docx]

**Supplementary Fig 1. The tumor immune microenvironment changes following neoadjuvant chemotherapy**

**(a)** Principal component analysis (PCA) of transcriptomic profiles from pre-treatment and post-treatment tumor samples. Each dot represents an individual sample.

**(b)** Unsupervised hierarchical clustering of differentially expressed genes (log2FC| ≥1 and FDR <0.05) between pre-treatment and post-treatment tumor samples. Rows represent genes, columns represent individual samples. Color scale indicates row‑scaled expression levels (red: upregulated, blue: downregulated).

**(c)** Volcano plot showing differentially expressed genes between pre-treatment and post-treatment tumor samples. Red and blue dots represent significantly upregulated and downregulated genes, respectively (|log₂FC| ≥ 1, P < 0.05). Gray dots indicate non‑significant genes.

**(d–e)** Gene set enrichment analysis (GSEA) of immune‑related signaling pathways in non‑matched tissues (d) and paired samples (e, n = 19). Statistical significance was determined by two-tailed unpaired Student's t-test, with P values indicated in the figure.

**(f)** The comparison of major immune cell populations between pre-treatment and post-treatment samples.

**Supplementary Fig 2. Impact of Chemotherapy Regimens and Treatment Cycles on Post-Neoadjuvant Tumor Biology**

**(a**) Comparative pathway enrichment analysis of post‑neoadjuvant chemotherapy (NACT) tumor specimens between patients treated with nab‑paclitaxel/carboplatin (n = 33) and conventional paclitaxel/carboplatin (n = 23). Normalized enrichment scores (NES) for selected pathways are shown; positive NES indicates enrichment in the nab‑paclitaxel group.

**(b-c)** Comparative analysis of pathway enrichment in post-NACT samples between 3 treatment cycles (n=47) versus 1-2 cycles (b, n=5) and 4-5 cycles (c, n=4). Representative pathways significantly altered between cycle groups are displayed.

**Supplementary Fig 3. The proteogenomic and metabolomics diversity following neoadjuvant chemotherapy**

**(a)** Principal component analysis (PCA) of proteomic and metabolomic profiles from pre-treatment and post-treatment tumor samples (n = 12 paired samples). Each dot represents an individual sample.

**(b)** Volcano plot displaying differentially expressed proteins between pre-treatment and post-treatment tumor samples (n = 12 paired samples). Red and blue dots represent significantly upregulated (n = 183) and downregulated (n = 97) proteins, respectively (|log₂FC| > 1.5, P < 0.05). Black dots indicate proteins with no significant change.

**(c)** Functional pathway enrichment analysis of upregulated (red) and downregulated (blue) proteins. Selected enriched pathways are shown.

**(d)** Volcano plot displaying differentially expressed metabolites between pre-treatment and post-treatment tumor samples (n = 12 paired samples). Red and blue dots represent significantly upregulated and downregulated metabolites, respectively (|log₂FC| > 1.5, P < 0.05). Black dots indicate metabolites with no significant change.

**(e)** Functional pathway enrichment analysis of differentially expressed metabolites. Top enriched metabolic pathways are displayed based on Kyoto Encyclopedia of Genes and Genomes (KEGG) annotations.

**(f)** Integrative analysis of proteogenomic and metabolomic alterations following neoadjuvant chemotherapy. Circos plot (or network diagram) showing correlations between significantly altered proteins and metabolites. Red lines indicate positive correlations; blue lines indicate negative correlations. Line thickness represents correlation strength (|r| > 0.5, P < 0.05).

FC, fold change; KEGG, Kyoto Encyclopedia of Genes and Genomes; PCA, principal component analysis.

**Supplementary Fig 4. The specific characteristics between pre-treated tumors with high and low CRS**

**(a)** TCGA molecular subtype distribution in non‑paired pre‑treatment (n = 27) and post‑treatment (n = 19) tumor samples stratified by CRS (high vs. low).

**(b)** Association between BRCA mutational status and CRS in pre‑treatment tumor samples. Stacked bar chart showing the proportion of BRCA wild‑type and mutant cases in CRS‑high versus CRS‑low groups. P value was calculated by two‑sided Fisher's exact test.

**(c)** Unsupervised hierarchical clustering of differentially expressed genes (DEGs, P < 0.05, |log₂FC| ≥ 1) between pre‑treatment tumors with high CRS and low CRS. Rows represent individual samples, columns represent genes. Color scale indicates row‑scaled expression levels (red: high expression; blue: low expression). Selected clusters of genes with distinct expression patterns are highlighted.

CRS, chemotherapy response score; TCGA, The Cancer Genome Atlas; FC, fold change; DEGs, differentially expressed genes.

**Supplementary Fig 5.** Functional pathway enrichment in platinum resistant (a, n=3) and sensitive (b, n=24) tumor samples.

**Supplementary Fig 6.** **Immune-related gene expression profiles in pre-treatment tumors stratified by chemotherapy response score.**

**(a)** Comparative analysis of chemokine, cytokine, and interleukin-related gene expression between pre-treatment tumors with high CRS (n = 17) and low CRS (n =10). Red indicates low CRS; blue indicates high CRS. Data are presented as mean ± SEM. * P<0.05, ** P<0.01, *** P<0.005 by two-tailed unpaired Student's t‑test.

**(b–c)** Comparative analysis of immunoglobulin gene expression between pre-treatment tumors with high and low CRS. Comparison analysis displaying expression levels of immunoglobulin heavy chain genes (b) and light chain genes (c). Data are presented as mean ± SEM. Statistical significance was determined by two-tailed unpaired Student's t-test, with P values indicated in the figure.

CRS, chemotherapy response score.

**Supplementary Fig 7.** **Carboplatin-resistant ovarian cancer cells exhibit enhanced mitochondrial oxidative phosphorylation activity.**

**(a)** Representative dose–response curves of six ovarian cancer cell lines following 48 h treatment with carboplatin (6.25–800 μM). Cell viability was normalized to vehicle‑treated controls. Data are presented as mean ± SD (n = 4 per group). The half‑maximal inhibitory concentration (IC₅₀) values were calculated for each cell line: OVCA‑433 (140.6 μM), HEY (145.2 μM), and OVCA‑R8 (103.0 μM) were classified as carboplatin‑resistant; SKOV3 (100.2 μM), OVCA‑429 (97.6 μM), and OVCA‑R3 (70.96 μM) were classified as carboplatin‑sensitive.

**(b)** Representative oxygen consumption rate (OCR) profiles over time in the six ovarian cancer cell lines. OCR was measured using a Seahorse XF analyzer and normalized to total protein levels. Data are presented as mean ± SD (n=5 parallel wells from a representative experiment).

**(c)** Comparison of OCR/ECAR ratio between carboplatin‑sensitive and carboplatin‑resistant ovarian cancer cell lines. Data are presented as mean ± SD (n = 4 per group). *P < 0.05, **P < 0.01 by two‑tailed unpaired Student's t‑test.

**(d)** Glutathione (GSH) levels in carboplatin‑sensitive versus carboplatin‑resistant ovarian cancer cell lines. Data are presented as mean ± SD (n = 3 per group). *P < 0.05, **P < 0.01 by two‑tailed unpaired Student's t‑test.

**(e)** Correlation between carboplatin IC₅₀ values and reactive oxygen species (ROS) levels in the six ovarian cancer cell lines. Spearman correlation coefficient (r) and P value are indicated (n=6).

OCR, oxygen consumption rate; ECAR, extracellular acidification rate; GSH, glutathione; ROS, reactive oxygen species; SD, standard deviation.

**Supplementary Fig 8.** **Representative carboplatin dose–response curves of six ovarian cancer patient‑derived organoids.**
Cells were treated with increasing concentrations of carboplatin for 48 h. Viability was normalized to vehicle‑treated controls. Data are presented as mean ± SEM from three independent experiments. Based on the calculated half‑maximal inhibitory concentration (IC₅₀) values, models were classified as carboplatin‑sensitive or carboplatin‑resistant.

**Supplementary Fig 9.** **Mitochondrial Complex I inhibition by IACS‑010759 disrupts mitochondrial homeostasis and reverses carboplatin resistance in ovarian cancer cells.**

**(a)** Representative oxygen consumption rate (OCR) profiles over time in OVCA‑433 and HEY cells treated with carboplatin, IACS‑010759 (mitochondrial Complex I inhibitor), or their combination. OCR was measured using a Seahorse XF analyzer and normalized to total protein levels. Data are presented as mean ± SD (n=8 parallel wells from a representative experiment).

**(b)** NAD⁺/NADH ratio in OVCA‑433 and HEY cells following treatment with carboplatin, IACS‑010759, or the combination. Data are presented as mean ± SD (n = 3 per group). **P < 0.01, ***P < 0.005, ****P < 0.001, by two-tailed unpaired Student's t-test.

**(c)** Representative transmission electron microscopy (TEM) images showing mitochondrial morphology in OVCA‑433 and HEY cells after the treatments of carboplatin, IACS‑010759, or the combination. Scale bar = 0.5 μm.

**(d)** Glutathione (GSH) levels in OVCA‑433 and HEY cells treated with carboplatin, IACS‑010759, or the combination. Data are presented as mean ± SD (n = 3 per group). ****P < 0.001 by two-tailed unpaired Student's t-test.

**(e)** Representative images (left) and statistical quantification of reactive oxygen species (ROS) levels in OVCA‑433 and HEY cells following indicated treatments. Data are presented as mean ± SD (n = 3 per group). ****P < 0.001 by two-tailed unpaired Student's t-test.

**(f)** Transcriptomic pathway enrichment analysis of OVCA‑433 and HEY cells treated with carboplatin versus the combination (DEGs, |log₂FC| > 1, P<0.05, n=3 per group). Red indicates upregulated pathways; green indicates downregulated pathways. Normalized enrichment score (NES) is indicated.

**(g)** Representative images (left) and statistical quantification of the Tail Moment (right) in the Alkaline Comet Assay. Cells were treated with Carboplatin (Cabo), Combi (Complex I inhibitor + Cabo), or Combi+CQ for 24h. Data are presented as mean ± SD (n=3 per group). **P < 0.01, ****P < 0.001 by two-tailed unpaired Student's t-test.

**(h)** Western blot analysis of ATR, p-ATR (Ser428), and RAD51 expression. OVCA-433 and HEY cells were treated as indicated. Tubulin was used as the loading control.

**(i)** Western blot analysis of γ-H2AX, Bcl-2, and Bax expression. Cells were treated with Cabo, Combi, or Combi+CQ. Tubulin was used as the loading control.

OCR, oxygen consumption rate; TEM, transmission electron microscopy; GSH, glutathione; ROS, reactive oxygen species; GSEA, gene set enrichment analysis; NES, normalized enrichment score; SD, standard deviation.

**Supplementary Fig 10.** **Knockdown of NDUFA8, a key subunit of mitochondrial Complex I, impairs mitochondrial bioenergetics.**

**(a–b)** Representative dose–response curves of OVCA433 (a) and HEY (b) cells transfected with non‑targeting siRNA or siRNAs targeting genes of mitochondrial Complex I–V. Cell viability was normalized to non‑targeting siRNA‑treated controls. Data are presented as mean ± SD (n=3 per group) with P value by two‑way ANOVA followed by Tukey's post hoc test. IC₅₀ values are shown in the upper right corner of each panel.

**(c–e)** Transcriptomic analysis of HEY and OVCA‑433 cells following NDUFA8 knockdown. (c) Heatmap showing differentially expressed genes (DEGs, |log₂FC| > 1, P<0.05, n=3 per group) between si‑NDUFA8 and Ctrl groups. Rows represent individual samples; columns represent genes. Color scale indicates row‑scaled expression levels (red: upregulated; blue: downregulated). (d–e) Gene Ontology (GO) enrichment analysis of upregulated (d) and downregulated (e) DEGs. Dot size represents gene count; color intensity indicates statistical significance (−log₁₀P value).

**(f–h)** Mitochondrial functional changes following NDUFA8 knockdown. (f) Mitochondrial membrane potential (MMP), (g) reactive oxygen species (ROS) levels, and (h) intracellular ATP levels in OVCA433 and HEY cells transfected with Ctrl or si‑NDUFA8. Data are presented as mean ± SD (n=3 per group). **P < 0.01 , ****P < 0.001 by two‑tailed unpaired Student's t‑test.

DEGs, differentially expressed genes; FC, fold change; GO, Gene Ontology; MMP, mitochondrial membrane potential; ROS, reactive oxygen species; SD, standard deviation.

**Supplementary Fig 11. CD 19^+^ B cells and NDUFA8 correlate with sensitivity to chemotherapy.**

**(a–b)** Immunohistochemical (IHC) staining of mitochondrial Complex subunits (NDUFA1, SDHB, and ATP5O) in platinum‑sensitive and platinum‑resistant xenograft tumors. (a) Representative IHC images. Scale bar = 100 μm. (b) Quantitative analysis of staining intensity. Data are presented as mean ± SD (n = 5 per group). ***P < 0.005 by two‑tailed unpaired Student's t‑test.

**(c)** Correlation analysis between CD19⁺ B cell infiltration and platinum sensitivity, stratified by KELIM score (>1 indicates sensitive; ≤1 indicates resistant). Each dot represents an individual patient sample (n = 61). **P < 0.01 by two‑tailed unpaired Student's t‑test.

**(d)** Correlation analysis between NDUFA8 expression and platinum sensitivity, stratified by KELIM score (>1 indicates sensitive; ≤1 indicates resistant). Each dot represents an individual patient sample (n = 65). **P < 0.01 by two‑tailed unpaired Student's t‑test.

IHC, immunohistochemical; SD, standard deviation; KELIM, CA‑125 elimination rate constant K (KELIM) index.

**Supplementary Table1. Multivariable Cox proportional hazards analysis evaluating independent predictors of chemotherapy response**

|  |  | **Univariate COX regression** | | | | | | | **Multivariate COX regression** | | | | | | |
| --- | --- | --- | --- | --- | --- | --- | --- | --- | --- | --- | --- | --- | --- | --- | --- |
|  |  | **HR** | **L95CI** | **H95CI** | **Coef** | **SE** | **Z** | **P value** | **HR** | **L95CI** | **H95CI** | **Coef** | **SE** | **Z** | **P value** |
| Age |  | 0.967 | 0.916 | 1.019 | -0.034 | 0.027 | -1.254 | 0.210 |  |  |  |  |  |  |  |
| FIGO | III | 0.835 | 0.300 | 2.318 | -0.181 | 0.521 | -0.347 | 0.729 |  |  |  |  |  |  |  |
|  | IV |  |  |  |  |  |  |  |  |  |  |  |  |  |  |
| NACT Strategy | TC |  |  |  |  |  |  |  |  |  |  |  |  |  |  |
|  | Nab TC | 0.634 | 0.081 | 4.994 | -0.455 | 1.053 | -0.433 | 0.665 |  |  |  |  |  |  |  |
| Times of NACT |  | 0.980 | 0.642 | 1.494 | -0.021 | 0.215 | -0.096 | 0.924 |  |  |  |  |  |  |  |
| BRCA | Wild type |  |  |  |  |  |  |  |  |  |  |  |  |  |  |
|  | Mutate | 0.401 | 0.141 | 1.139 | -0.913 | 0.532 | -1.715 | 0.086 |  |  |  |  |  |  |  |
| HRD | HRD |  |  |  |  |  |  |  |  |  |  |  |  |  |  |
|  | HRP | 1.834 | 0.624 | 5.395 | 0.607 | 0.550 | 1.102 | 0.270 |  |  |  |  |  |  |  |
| CRSlevel | High |  |  |  |  |  |  |  |  |  |  |  |  |  |  |
|  | Low | 2.039 | 0.720 | 5.770 | 0.712 | 0.531 | 1.342 | 0.180 |  |  |  |  |  |  |  |
| KELIM | <1 |  |  |  |  |  |  |  |  |  |  |  |  |  |  |
|  | >1 | 0.092 | 0.013 | 0.651 | -2.385 | 0.998 | -2.391 | **0.017** | 0.138 | 0.021 | 0.899 | -1.977 | 0.954 | -2.072 | **0.038** |
| CD19+B cells |  | >20 | >20 | >20 | 70.6 | 28.9 | 2.44 | **0.015** | >20 | 0.896 | >20 | 65.1 | 33.3 | 1.96 | **0.049** |

**Supplementary table 2**
